# Effect of Intensivist Communication in a Simulated Setting on Interpretation of Prognosis Among Family Members of Patients at High Risk of Intensive Care Unit Admission: A Randomized Trial

**DOI:** 10.1001/jamanetworkopen.2020.1945

**Published:** 2020-04-01

**Authors:** Ian M. Oppenheim, Emma M. Lee, Scott T. Vasher, Sandra E. Zaeh, Joanna L. Hart, Alison E. Turnbull

**Affiliations:** 1 Division of Pulmonary and Critical Care Medicine, Johns Hopkins University School of Medicine, Baltimore, Maryland; 2 Department of Internal Medicine, Johns Hopkins University School of Medicine, Baltimore, Maryland; 3 Division of Pulmonary, Allergy, and Critical Care Medicine, University of Pennsylvania, Philadelphia; 4 Leonard Davis Institute of Health Economics, University of Pennsylvania, Philadelphia; 5 Palliative and Advanced Illness Research Center, Perelman School of Medicine, University of Pennsylvania, Philadelphia; 6 Department of Epidemiology, Johns Hopkins Bloomberg School of Public Health, Baltimore, Maryland; 7 Outcomes After Critical Illness and Surgery Group, Johns Hopkins University School of Medicine, Baltimore, Maryland

## Abstract

**Importance:**

Discordance about prognosis between a patient’s health care decision-making surrogate and the treating intensivist is common in the intensive care unit (ICU). Empowering families, friends, and caregivers of patients who are critically ill to make informed decisions about care is important, but it is unclear how best to communicate prognostic information to surrogates when a patient is expected to die.

**Objective:**

To determine whether family members, who are often health care decision-making surrogates, interpret intensivists as being more optimistic when questions about prognosis in the ICU are answered indirectly.

**Design, Setting, and Participants:**

This web-based randomized trial was conducted between September 27, 2019, and October 17, 2019, among a national sample of adult children, spouses, partners, or siblings of people with chronic obstructive pulmonary disease who were receiving long-term oxygen therapy. Participants were shown video vignettes depicting an intensivist answering a standardized question about the prognosis of a patient at high risk of death on day 3 of ICU admission. Participants were excluded if they had worked as a physician, nurse, or advanced health care practitioner. Data were analyzed from October 18, 2019, to November 12, 2019.

**Interventions:**

Participants were randomized to view 1 of 4 intensivist communication styles in response to the question “What do you think is most likely to happen?”: (1) a direct response (control), (2) an indirect response comparing the patient’s condition with that of other patients, (3) an indirect response describing the patient’s deteriorating physiological condition, or (4) redirection to a discussion of the patient’s values and goals.

**Main Outcomes and Measures:**

Participant responses to 2 questions: (1) “If you had to guess, what do you think the doctor thinks is the chance that your loved one will survive this hospitalization?” and (2) “What do you think are the chances that your loved one will survive this hospitalization?” answered using a 0% to 100% probability scale.

**Results:**

Among 302 participants (median [interquartile range] age, 49 [38-59] years; 204 [68%] women) included in the trial, 165 (55%) were adult children of the individual with chronic obstructive pulmonary disease; 77 participants were randomized to view a direct response, 77 participants were randomized to view an indirect response referencing other patients, 68 participants were randomized to view an indirect response referencing physiological condition, and 80 participants were randomized to view a redirection response. Compared with participants who viewed a direct response, participants who viewed an indirect response referencing other patients (β = 10 [95% CI, 1-19]; *P* = .03), physiological condition (β = 10 [95% CI, 0-19]; *P* = .04), or redirection to a discussion of the patient’s values and goals (β = 19 [95% CI, 10-28]; *P* < .001) perceived the intensivist to have a significantly more optimistic prognostic estimate.

**Conclusions and Relevance:**

These findings suggest that family members interpret indirect or redirection responses to questions about prognosis in the ICU setting as more optimistic than direct responses.

**Trial Registration:**

ClinicalTrials.gov Identifier: NCT04239209

## Key Points

**Question:**

Do intensivist communication patterns affect the way family members understand their loved one’s prognosis in the intensive care unit?

**Findings:**

In this randomized trial of 302 family members of people with chronic obstructive pulmonary disease receiving long-term oxygen therapy, participants were asked to imagine their family member was in the intensive care unit, and the participants were presented video vignettes of an intensivist who expected a patient to die answering the prognostic question “What do you think is most likely to happen?” Participants who viewed videos of the intensivist using indirect or redirection language perceived the intensivist to be more optimistic than participants who viewed a video of the intensivist answering the question directly.

**Meaning:**

These findings suggest that family members interpret physicians’ indirect responses to questions about prognosis as more optimistic than direct responses.

## Teaser

This randomized trial examines the effects of 4 types of intensivist responses to the question “What do you think is most likely to happen?” among family members of people with chronic obstructive pulmonary disease in a hypothetical intensive care unit setting.

## Introduction

Many individuals in the US near the end of life prefer to forgo the use of life support,^1,2,3^ yet high rates of intensive care unit (ICU) admission and mechanical ventilation in the last month of life persist.^4,5,6^ Recognizing this discrepancy, the American Board of Internal Medicine Foundation’s Choosing Wisely campaign endorsed the following recommendation for critical care clinicians in 2014: “Don’t continue life support for patients at high risk for death or severely impaired functional recovery without offering patients and their families the alternative of care focused entirely on comfort.”^7^ Because critical illness prevents many patients from communicating, family members or caregivers act as surrogate decision-makers for most patients in the ICU.^8^ Effective communication about prognosis with these surrogates is essential to ensuring they make informed decisions about care.^9^

Previous trials have tested methods for communicating prognosis to surrogates in ICU settings,^10,11^ and substantial surrogate-intensivist discordance about prognosis remains^10,12,13^ even when surrogates rate the quality of physician communication highly.^14^ Surrogate-intensivist discordance about prognosis is caused by surrogate misunderstanding and differences between surrogate and intensivist beliefs about prognosis, and discordance tends to result in families holding more optimistic expectations in general.^12,15,16^ Previous research on communicating prognosis has enrolled people actively acting as surrogates in ICU settings. Anxiety, depression, sleep deprivation, anticipatory grief, and posttraumatic stress are common in this population^17,18,19,20^ and likely affect how they interpret prognostic information.^17,20,21^

We hypothesize that the complex language used by intensivists and the acute stress and exhaustion of being a surrogate in an ICU setting contribute to surrogate misunderstandings. If this hypothesis is correct, direct communication about prognosis using simple language has the potential to minimize misunderstandings and reduce discordant surrogate optimism. However, under this hypothesis, discordant optimism attributable to differences in beliefs should not be affected by the language a physician uses to convey prognosis. Therefore, we designed a fully online randomized trial to study communication of prognosis to close family members of adults with chronic obstructive pulmonary disease (COPD) who require long-term home oxygen therapy (LTOT). These family members are likely to become surrogate decision-makers in the near future owing to the severity of the patient’s illness^22,23^ but are unlikely to be acutely stressed about the immediate survival of their family member while participating in the study at home. We hypothesized that this population would perceive intensivists to be less optimistic (and thus experience less misunderstanding) when a question about the prognosis of a patient who was critically ill was answered directly, but that differences in belief about prognosis would be unaffected by an intensivist’s communication style.

## Methods

We conducted a web-based, parallel-group, randomized trial to assess the effect of intensivist communication patterns on prognosis interpretation. The trial protocol is available in Supplement 1. This trial was not prospectively registered at ClinicalTrials.gov because it examined simulated health outcomes only. It was retrospectively registered on January 22, 2020. The study was designated as exempt from review by the Johns Hopkins Medicine institutional review board. All participants provided consent to participate in the study. The study was conducted between September 27, 2019, and October 17, 2019, in the US. Our reporting follows the Consolidated Standards of Reporting Trials (CONSORT) reporting guidelines.

### Recruitment

Study participants were recruited by Qualtrics, an internet survey company, and the trial was performed using the Qualtrics online survey platform. Qualtrics maintains a roster of millions of US residents who provide background information on their eligibility for surveys and studies. Online recruitment via research tools, such as Qualtrics and Amazon.com’s Mechanical Turk, generates sample responses that are largely comparable to those collected via conventional methods.^24^ Adults within the Qualtrics roster who had previously reported having a family member who was chronically ill were screened to participate in this study. This method of recruitment has successfully been used to recruit families of patients recently treated in an ICU for studies of medical communication.^25,26^ Participants consented to participate online before beginning the trial and were compensated for study completion.

### Eligibility, Randomization, and Primary Exposure

Study eligibility criteria included being aged 18 years or older and being the spouse, partner, adult child, or sibling of an adult who was currently living with COPD and receiving LTOT. We excluded participants who reported ever having worked as a physician, nurse, or advanced health care practitioner. Participants were randomly assigned in a 1-to-1-to-1-to-1 ratio to view a video depicting 1 of 4 emblematic ways intensivists answered a surrogate’s scripted prognostic question “What do you think is most likely to happen?” during a simulated ICU family meeting.^27^ The allocation sequence used a computer-based randomization tool integrated into the survey platform that allowed for equal likelihood of assignment to 1 of 4 groups. The responses were verbatim replications of intensivists’ actual responses recorded as part of a previous randomized clinical trial conducted in a high-fidelity simulation center.^28^ All responses came from intensivists who did not expect the hypothetical patient to survive hospitalization and who each reported after the simulation that they had conveyed prognosis for risk of death. We refilmed a single intensivist delivering each response to standardize intensivist demographic characteristics, accent, and body language and to protect the identity of intensivists participating in the original trial. The refilmed communication patterns were (1) a direct response in which the intensivist acknowledges that he is not certain but believes the patient will not survive hospitalization, (2) an indirect response describing the outcomes of other people similar to the patient in question, (3) an indirect response describing the deteriorating physiological condition of the patient, and (4) redirection to a conversation about the values of the patient and possible future decisions (Table 1 and Video).

**Table 1.** Intensivist Responses to the Question “What do you think is most likely to happen?”

Response type	Quotation
Direct (28 words, 12 s)	“I think the most likely thing, which doesn’t mean it’s the certain thing, I think the most likely thing is that he’s not going to survive this hospitalization.”
Indirect by describing other patients (17 words, 8 s)	“Many people like your father that we’ve cared for, they do not survive this type of illness.”
Indirect by describing physiological condition (133 words, 45 s)	“What I’m very concerned about is given the amount of oxygen that he requires on the mechanical ventilator… I mean, if his oxygen requirements continue to go up and up and up and the antibiotics don’t kick in, ...given his age... given his medical history, there is certainly a possibility that ultimately we may get to a point where the mechanical ventilator is not able to deliver the amount of oxygen required for him to keep his organs going. And these are just things that... we are not there yet; however, every single day, I’m going to be reassessing him, so I’m really concerned that he might not... we might not be able to deliver the amount of support to keep his organs alive and they may just continue to shut down.”
Redirection (148 words, 52 s)	“So, some of it is... and you know... in no way do we need to make any decisions today, but there is a likelihood that there is a significant possibility that we’ll be in a place where we might have to have some tough conversations and make some tough decisions, okay? And some of that goes to... you know... our job, since he can’t speak for himself since you’re his next of kin... his closest family member, is to try and make the decision that he would make if he could speak for himself, which is oftentimes hard because that’s not always the decision that we want to make as the loved one, you know? My experience is we want to hold on, but the question isn’t that. The question is would he want that? And some of that centers around what did he consider quality of life?”

**Video.** Intensive Care Unit Prognosis Communication Options Intensivist responses were categorized in the following ways: 1. Direct response: acknowledges uncertainty but believes the patient will not survive hospitalization. 2. Indirect response: describes the prognosis of other people similar to the patient in question. 3. Indirect response: describes the deteriorating physiological condition and potential future problems. 4. Redirection: discusses the values of the patient and possible future decisions.

Before viewing the assigned video, participants were instructed to imagine that their family member with COPD who was receiving LTOT had been in an ICU for 3 days and that the intensivist in the video was speaking directly to the study participant. They were further instructed to imagine that they had just asked “What do you think is most likely to happen?” and that the video displayed the intensivist’s response. Immediately after viewing the video, participants were asked 2 previously validated questions to measure their belief about their family member’s chances of surviving the hospitalization on a 0% to 100% scale and their perception of the intensivist’s belief about their family member’s chances of survival.^10,12^ To ensure all participants received the same amount of the exposure, the survey prevented participants from advancing to the next screen until the video had finished playing, and participants were unable to navigate back to the video page after advancing. To help ensure high-quality responses, a question about demographic data was repeated at 2 points. Questionnaires that did not include a consistent response to the repeated question, were incomplete, or were completed in less than 7 minutes or more than 60 minutes were not analyzed.

### Outcomes

Outcomes were determined prior to data collection. The primary outcome was the participant’s perception of the intensivist’s prognostic estimate, evaluated as the response to the question, “If you had to guess, what do you think the doctor thinks is the chance that your loved one will survive this hospitalization?” answered using a 0% to 100% probability scale^12^ anchored at each extreme with the phrases “no chance of survival” and “will definitely survive.” Secondary outcomes included (1) the participant’s prognostic estimate, evaluated as the response to the question “What do you think are the chances that your loved one will survive this hospitalization?” assessed using the same scale, (2) respondent difference in belief (eFigure 1 in Supplement 2), (3) respondent confidence that they understood the intensivist’s belief about prognosis, and (4) respondent confidence in their own prognostic estimate. Difference in belief was defined as a participant’s prognostic estimate minus their perception of the physician’s prognostic estimate.^12^ Both questions about confidence were assessed using a 5-point Likert scale. We also collected information on demographic characteristics including age, sex, race, ethnicity, relationship to the family member with COPD, whether their family member with COPD had ever required a ventilator during a hospitalization, formal education level, census bureau–designated region of residence, health numeracy as assessed by an 8-item Rasch-based numeracy scale,^29^ trust in the intensivist depicted in the video using the Wake Forest Physician Trust Scale,^30^ and health literacy assessed by the Short Test of Functional Health Literacy in Adults.^31,32^

### Statistical Analysis

Based on surrogate decision-makers’ interpretations of prognostic information in previous research,^16^ we estimated that enrolling 75 participants in each group of the trial would provide power of 0.9 to detect at least an 8-point difference in the mean interpretation of intensivist statements assuming a 2-sided α of .05 and an estimated SD of 15. Participant characteristics were reported using descriptive statistics and responses to continuous outcome measures were visualized using kernel density plots. Multivariable linear regression was used to estimate the effect of communication pattern on (1) participant perception of the intensivist’s prognostic estimate, (2) the participant’s prognostic estimate, and (3) difference in belief. A direct communication pattern was treated as the comparator (control group) in all models. All models were adjusted for a priori hypothesized confounders, including the relationship of the participant to the family member with COPD, whether the participant’s family member had experienced mechanical ventilation, education level, numeracy, the Wake Forest Physician Trust Scale score, and health literacy score. Residual plots were reviewed to evaluate model assumptions. Participants who selected confident or very confident on the 5-point Likert scale were analyzed as being confident. The difference in proportion of confident participants was estimated using the sample proportions in each group compared to the control group. The null hypothesis of no difference was tested using a Fisher exact test. A sensitivity analysis was performed to ensure estimates were robust to the threshold chosen for confidence (eg, including neutral as a confident response). A *P* value of .05 was treated as statistically significant. Analyses were performed using R statistical software version 3.6.1 (R Project for Statistical Computing). Data were analyzed from October 18, 2019, to November 12, 2019.

## Results

### Participants

A total of 8911 US adults with adult family members who were chronically ill were screened for eligibility. We excluded 5990 adults because their family member did not have COPD, 1945 adults because they had worked as a physician, nurse, or advanced health care practitioner, 537 adults because their family member with COPD did not require LTOT, and 71 adults because their responses did not pass quality control checks (eFigure 2 in Supplement 2). Of 368 eligible participants, 66 were excluded because they did not complete the study; therefore, 302 participants were included in analysis. Median (interquartile range [IQR]) participant age was 49 (38-59) years, 204 participants (68%) were women, and 268 participants (89%) were white. Among 302 participants, 165 participants (55%) were the adult children of individuals with COPD who were receiving LTOT, while 107 participants (35%) were spouses or partners, and 28 participants (9%) were siblings. There were 171 participants (57%) who reported that their family member had required a ventilator (ie, breathing machine) during a previous inpatient hospitalization (Table 2). Among 302 included participants, 77 were randomized to view a direct response, 77 were randomized to view an indirect response referencing other patients, 68 were randomized to view an indirect response referencing physiological condition, and 80 were randomized to view a redirection response.

**Table 2.** Trial Participant Characteristics

Characteristic	No. (%) (N = 302)^a^
Age, median (IQR), y	49 (38-59)
Women	204 (68)
Race	
White	268 (89)
Black	15 (5)
Other or multiracial	14 (5)
Prefer not to answer	5 (2)
Ethnicity	
Hispanic or Latino	27 (9)
Relationship to patient	
Adult child	165 (55)
Spouse or partner	109 (36)
Sibling	28 (9)
Has your loved one ever needed a breathing machine (ventilator) during a hospital stay?	
Yes	171 (57)
No	108 (36)
Unsure	23 (8)
Education	
<High school	11 (4)
High school or GED	86 (28)
Some college	108 (36)
4-y degree	70 (23)
Graduate or professional degree	27 (9)
Census region of residence	
South	132 (44)
Midwest	79 (26)
Northeast	54 (18)
West	37 (12)
Numeracy score, median (IQR)^b^	5 (3-6)
Wake Forest Physician Trust Scale score, median (IQR)^c^	18 (14-20)
Functional Health Literacy score, median (IQR)^d^	35 (32-36)

Abbreviations: GED, general education diploma; IQR, interquartile range.

^a^ Due to rounding, not all percentages total to 100.

^b^ Range, 0 to 8; higher numbers indicate greater numeracy.

^c^ Focused on the intensivist depicted in the study videos (range, 5-25; higher numbers indicate greater trust).

^d^ Range, 0 to 36; higher numbers indicate greater health literacy.

### Primary Outcome

**Figure 1.**
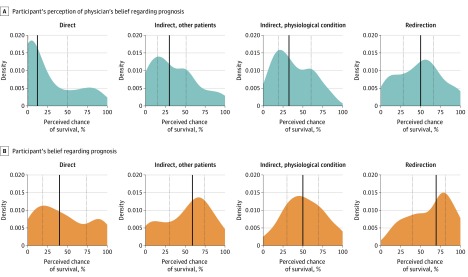
Comparison of Prognostic Estimates Among Family Members Stratified by Intensivist Response A and B, Density plots (smoothed, continuous versions of histograms estimated from the data) illustrate participants’ prognostic estimates for their family member with chronic obstructive pulmonary disease when answering the questions “If you had to guess, what do you think the doctor thinks is the chance that your loved one will survive this hospitalization?” (A) and “What do you think are the chances that your loved one will survive this hospitalization?“ (B) after viewing a video-recorded response of an intensivist answering the question “What do you think is most likely to happen?” during an intensive care unit family meeting. Participants were randomized to view 1 of 4 responses: direct, indirect referring to other patients, indirect referring to deteriorating physiological condition, and redirection to a discussion of the patient’s values. Solid black vertical lines indicate median value; dotted lines, interquartile range.

Participants who viewed an indirect response referencing outcomes of other patients perceived the intensivist to have a significantly more optimistic prognostic estimate compared with participants who viewed a direct response about prognosis (β = 10 [95% CI, 1-19]; *P* = .03). The same was found for participants who viewed an indirect response explaining deteriorating physiological conditions (β = 10 [95% CI, 0-19]; *P* = .04) and those who viewed a response that redirected the conversation to a discussion of patient goals and values (β = 19 [95% CI, 10-28]; *P* < .001) compared with participants who viewed a direct response about prognosis (Table 3 and Figure 1; eTable 1 in Supplement 2).

**Table 3.** Effect of Intensivist’s Response on Family Member Perception of the Intensivist’s Prognostic Estimate, Belief About Prognosis, and Difference in Belief

Model	β (95% CI)	*P* value
**Question 1: “What do you think the doctor thinks is the chance that your loved one will survive this hospitalization?”**^a^**^,^**^b^		
Direct	[Reference]	NA
Indirect		
Other patients	10 (1 to 19)	.03
Physiological condition	10 (0 to 19)	.04
Redirection	19 (10 to 28)	<.001
**Question 2: “What do you think are the chances that your loved one will survive this hospitalization?”**^a^^,^^b^		
Direct	[Reference]	NA
Indirect		
Other patients	9 (0 to 18)	.06
Physiological condition	8 (−2 to 17)	.11
Redirection	19 (10 to 28)	<.001
**Difference in belief**^b^^,^^c^		
Direct	[Reference]	NA
Indirect		
Other patients	−1 (−8 to 5)	.72
Physiological condition	−2 (−9 to 5)	.59
Redirection	0 (−6 to 7)	.90

Abbreviation: NA, not applicable.

^a^ Measured on a 0-100 scale, with higher scores indicating a greater likelihood of survival.

^b^ Adjusted for participant’s relationship to patient, education, numeracy, trust, and patient’s prior experience with ventilation.

^c^ Calculated as response to question 2 minus response to question 1.

### Secondary Outcomes

**Figure 2.**
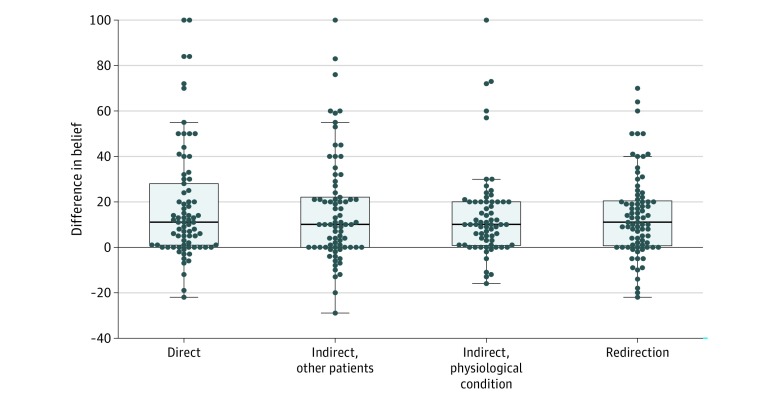
Difference in Belief Among Family Members Stratified by Intensivist Response Difference in belief was calculated as the participant’s prognostic estimate minus their perception of the intensivist’s prognostic estimate. Positive values indicate a more optimistic difference in belief, meaning the participant holds a higher prognostic estimate than what they perceive the intensivist to hold; negative values indicate that the participant is more pessimistic. A difference in belief of 0 indicates that there is no difference between the participant’s perception of the intensivist’s prognostic estimate and the participant’s own estimate. Dots indicate individual values; center lines, medians; boxes, interquartile ranges; and whiskers, largest and smallest values no further than 1.5-fold from the interquartile ranges.

There was no statistically significant difference in the participant’s own prognostic estimates among those who viewed any indirect response compared with those who viewed the direct response, but family members who viewed the redirection to patient wishes response had a mean of 19 percentage points more belief that their family member would recover (β = 19 [95% CI, 10-28]; *P* < .001) compared with patients who viewed the direct response (Table 3). Median difference in belief was positive and similar across all groups (Figure 2). Optimism (a positive difference in belief) was observed in 58 participants (75%) who viewed the direct communication pattern, 54 participants (79%) who viewed the indirect response referencing other patients, 54 participants (70%) who viewed the indirect response explaining deteriorating physiological conditions, and 60 participants (75%) who viewed the redirection response. Participants who viewed the direct response were equally as likely to feel confident that they knew what the doctor was thinking (52 participants [68%]) as participants who viewed an indirect response referencing other patients (48 participants [62%]; difference in proportions vs direct response, 5% [95% CI, –11% to 21%]; *P* = .61), participants who viewed an indirect response explaining deteriorating physiological conditions (43 participants [63%]; difference in proportions vs direct response, 4% [95% CI, –13% to 21%]; *P* = .71), or participants who viewed a redirection response (51 participants [64%]; difference in proportions vs direct response, 4% [95% CI, –12% to 20%]; *P* = .74) (eTable 2 in Supplement 2). Among participants who viewed the direct response, 59 (77%) were confident in their own understanding of prognosis, and the proportions of participants in the other trial groups who were confident were not statistically significantly different from the direct response (eTable 2 in Supplement 2). Results involving confidence did not change substantially in sensitivity analyses (eTable 3 in Supplement 2).

## Discussion

In this web-based randomized trial of intensivist responses to a family member’s question about a loved one who was critically ill, “What do you think is most likely to happen?” we found that family members of people with COPD perceived the intensivist to be significantly more optimistic when the intensivist provided an indirect response or redirected the conversation to a discussion of patient goals compared with when the intensivist provided a direct response. We also found that family members’ own prognostic estimates were more optimistic when they were not given a direct response, but this increase was only statistically significant when the intensivist redirected the conversation. Difference in belief (the difference between what the participant perceives the intensivist’s prognostic estimate to be and the participant’s own prognostic estimate) was optimistic for approximately three-fourths of participants regardless of intensivist response. Participants were also equally likely to be confident in their perception of intensivist prognostic estimates and their own prognostic estimate across intensivist responses.

The significant differences in participant perception of intensivist prognostic estimates suggests that common ways physicians answer questions about prognosis are not interchangeable or simply a matter of style. Speaking indirectly about the prognosis for a patient who is critically ill is commonly viewed as a way to communicate bad news without causing psychological harm. Our findings suggest that indirect statements about prognosis are not simply gentle versions of direct statements. Instead, they are interpreted by family members as evidence that the speaker is more optimistic about the patient’s outcome.

The distribution of difference in belief^12^ among family members remained remarkably similar across intensivist responses. Previously identified causes of optimistic difference in belief include religiosity, performative optimism (ie, needing to maintain hope for the patient’s benefit), belief that physicians are inherently pessimistic, and belief that the surrogate’s family member is better than average (ie, the Lake Wobegon effect).^12,16^ A key difference in our trial is that we enrolled family members who were not actively serving as ICU surrogates. This meant that participants were unlikely to be experiencing the psychiatric stressors common among ICU surrogates, such as anxiety, depression, and sleep deprivation, and the associated cognitive blunting.^17,18,20^ The persistence of optimistic difference in belief in our study population suggests that surrogate optimism may exist before a person who is chronically ill arrives in the ICU and is not primarily attributable to the stress of acting as a surrogate for a family member who is acutely ill.

Whether a family member’s optimism comes from misunderstanding or difference in belief, it can have profound effects on patient care. Optimism that is not aligned with the prognostic estimate of a physician has been associated with increased use of life support at the end of life.^33,34,35,36,37^ When realistic, optimism can be adaptive and is associated with positive health behaviors and better physical and mental health.^38,39^ However, unrealistic optimism has been shown to have detrimental mental and physical health effects^38,40^ in addition to possibly increasing the risk of errors in judgement when acting as a surrogate decision-maker.^41^

While our simulations portrayed a surrogate who clearly requested to know the physician’s thoughts about prognosis, some patients in the ICU and their family members prefer not to discuss prognosis. We recommend asking patients and their surrogates whether they wish to discuss prognosis and respecting their response. When a surrogate requests information about the prognosis of a patient whom physicians expect to die, our results indicate that direct and unambiguous language is the most effective way for intensivists to minimize misunderstanding. Two considerations are important to consider when using direct language. First, being direct is not synonymous with being certain. The recorded direct answer in this trial included the words: “I think the most likely thing, which doesn’t mean it’s the certain thing… is that he’s not going to survive this hospitalization” (Table 1). Second, our study was not designed to evaluate the effect of direct communication on the acute stress experienced by physicians or surrogates, trust within the surrogate-physician relationship, family member satisfaction, or decisions about patient care. All of these outcomes are areas that warrant future research.

Additionally, approximately two-thirds of family members were confident that they knew what the intensivist thought about prognosis, and approximately three-fourths of family members were confident that they understood their loved one’s prognosis regardless of the response viewed. This confirms that asking families to evaluate the clarity or completeness of communication during family meeting is unlikely to be fruitful, a finding previously seen in a study of ICU surrogate decision-makers.^14^ This finding also supports the importance of asking family members to explain what they understood in their own words, such as in the ask-tell-ask technique,^42^ when assessing patient and family understanding in the clinical setting.

### Limitations and Strengths

Our study has limitations. First, web-based randomized trials inherently restrict enrollment to people with internet access and computer or smartphone literacy. However, we found similar health literacy scores and physician trust scores compared with studies conducted in ICUs.^12^ Second, our sample included a disproportionately high number of white participants, limiting the generalizability of our findings and preventing us from making inferences about whether the observed relationship between communication style and interpretation varies across race. Third, participants in our trial may never become legal surrogate medical decision-makers for their family member with COPD; however, by limiting analyses to spouses, adult children, and siblings, we have ensured that all participants are immediate family members who are likely to be involved in decisions during times of critical illness. Fourth, by asking all participants to imagine they were viewing an intensivist on ICU day 3, we were not able to assess whether our findings apply to other settings (eg, the emergency department), or other time frames (eg, ICU day 10). Our findings apply to situations in which a patient in the ICU has a preexisting chronic condition and may not be applicable to acute critical illness in a previously healthy person. Fifth, we acknowledge that our trial was performed in a hypothetical context, and further studies in the ICU setting are warranted. Future studies using simulation may help to establish how clinicians can use framing to reduce prognostic discordance.

A strength of our study is the use of a single intensivist across all videos in the trial to ensure observed effects were attributable to word choice rather than intensivist demographic characteristics or accent. We also used verbatim responses from attending intensivists answering a standardized question about prognosis, which more accurately reflect how families learn prognostic information compared with written statements or idealized responses. An additional strength is that our trial examined family members of people with COPD, a population with early palliative care needs who tend to have poor prognostic awareness.^43^ Our findings may also be applicable to prognostic discussions between physicians and family caregivers of people with COPD in outpatient clinics.

## Conclusions

This study’s findings suggest that when asking questions about prognosis, family members perceived intensivists who gave indirect responses or redirected the conversation as being more optimistic than intensivists who answered their questions directly. Regardless of how clearly intensivists answered questions about prognosis, most family members were confident that they understood what the intensivist was thinking and remained optimistic about their loved one’s survival. These patterns existed even when families were not experiencing the acute stress of an ICU admission. When families request information about prognosis, intensivists should convey the clinical team’s view using a simple, direct response. However, intensivists should not assume that families will agree with them, no matter how carefully they choose their words.

## Notes

Supplement 1. **Trial Protocol**

Supplement 2. **eFigure 1.** Definition of Difference in Belief **eFigure 2.** Study Flow Diagram **eTable 1.** Unadjusted Models of the Effect of Intensivist’s Response on Family Member Understanding of the Intensivist, Belief About Prognosis, and Difference in Belief **eTable 2.** Effect of Intensivist’s Response on Family Member Confidence in Their Own Prognostic Estimate and Their Perceived Prognostic Estimate of the Intensivist **eTable 3.** Sensitivity Analysis of Effect of Intensivist’s Response on Family Member Confidence in Their Own Prognostic Estimate and Their Perceived Prognostic Estimate of the Intensivist **eReference.**

Supplement 3. **Data Sharing Statement**

## References

[1] Fischer S, Min S-J, Cervantes L, Kutner J Where do you want to spend your last days of life? low concordance between preferred and actual site of death among hospitalized adults. J Hosp Med. 2013;8(4):-. doi:10.1002/jhm.2018 23440934 PMC4705849

[2] Barnato AE, Herndon MB, Anthony DL, Are regional variations in end-of-life care intensity explained by patient preferences: a study of the US Medicare population. Med Care. 2007;45(5):386-393. doi:10.1097/01.mlr.0000255248.79308.41 17446824 PMC2147061

[3] Fried TR, Bradley EH, Towle VR, Allore H Understanding the treatment preferences of seriously ill patients. N Engl J Med. 2002;346(14):1061-1066. doi:10.1056/NEJMsa012528 11932474

[4] Angus DC, Barnato AE, Linde-Zwirble WT, ; Robert Wood Johnson Foundation ICU End-of-Life Peer Group Use of intensive care at the end of life in the United States: an epidemiologic study. Crit Care Med. 2004;32(3):638-643. doi:10.1097/01.CCM.0000114816.62331.08 15090940

[5] Teno JM, Gozalo P, Trivedi AN, Site of death, place of care, and health care transitions among US Medicare beneficiaries, 2000-2015. JAMA. 2018;320(3):264-271. doi:10.1001/jama.2018.8981 29946682 PMC6076888

[6] Teno JM, Gozalo P, Khandelwal N, Association of increasing use of mechanical ventilation among nursing home residents with advanced dementia and intensive care unit beds. JAMA Intern Med. 2016;176(12):1809-1816. doi:10.1001/jamainternmed.2016.5964 27723891 PMC5138104

[7] Halpern SD, Becker D, Curtis JR, ; Choosing Wisely Taskforce; American Thoracic Society; American Association of Critical-Care Nurses; Society of Critical Care Medicine An official American Thoracic Society/American Association of Critical-Care Nurses/American College of Chest Physicians/Society of Critical Care Medicine policy statement: the Choosing Wisely top 5 list in critical care medicine. Am J Respir Crit Care Med. 2014;190(7):818-826. doi:10.1164/rccm.201407-1317ST 25271745

[8] Silveira MJ, Kim SYH, Langa KM Advance directives and outcomes of surrogate decision making before death. N Engl J Med. 2010;362(13):1211-1218. doi:10.1056/NEJMsa0907901 20357283 PMC2880881

[9] Turnbull AE, Hartog CS Goal-concordant care in the ICU: a conceptual framework for future research. Intensive Care Med. 2017;43(12):1847-1849. doi:10.1007/s00134-017-4873-2 28656453 PMC5717114

[10] Lee Char SJ, Evans LR, Malvar GL, White DB A randomized trial of two methods to disclose prognosis to surrogate decision makers in intensive care units. Am J Respir Crit Care Med. 2010;182(7):905-909. doi:10.1164/rccm.201002-0262OC 20538959 PMC2970862

[11] Barnato AE, Arnold RM The effect of emotion and physician communication behaviors on surrogates’ life-sustaining treatment decisions: a randomized simulation experiment. Crit Care Med. 2013;41(7):1686-1691. doi:10.1097/CCM.0b013e31828a233d 23660727 PMC3687021

[12] White DB, Ernecoff N, Buddadhumaruk P, Prevalence of and factors related to discordance about prognosis between physicians and surrogate decision makers of critically ill patients. JAMA. 2016;315(19):2086-2094. doi:10.1001/jama.2016.5351 27187301

[13] Fried TR, Bradley EH, O’Leary J Prognosis communication in serious illness: perceptions of older patients, caregivers, and clinicians. J Am Geriatr Soc. 2003;51(10):1398-1403. doi:10.1046/j.1532-5415.2003.51457.x 14511159

[14] Chiarchiaro J, Buddadhumaruk P, Arnold RM, White DB Quality of communication in the ICU and surrogate’s understanding of prognosis. Crit Care Med. 2015;43(3):542-548. doi:10.1097/CCM.0000000000000719 25687030 PMC4336600

[15] Boyd EA, Lo B, Evans LR, “It’s not just what the doctor tells me”: factors that influence surrogate decision-makers’ perceptions of prognosis. Crit Care Med. 2010;38(5):1270-1275. doi:10.1097/CCM.0b013e3181d8a217 20228686 PMC3530838

[16] Zier LS, Sottile PD, Hong SY, Weissfield LA, White DB Surrogate decision makers’ interpretation of prognostic information: a mixed-methods study. Ann Intern Med. 2012;156(5):360-366. doi:10.7326/0003-4819-156-5-201203060-00008 22393131 PMC3530840

[17] Verceles AC, Corwin DS, Afshar M, Half of the family members of critically ill patients experience excessive daytime sleepiness. Intensive Care Med. 2014;40(8):1124-1131. doi:10.1007/s00134-014-3347-z 24898893 PMC4500523

[18] Pochard F, Azoulay E, Chevret S, ; French FAMIREA Group Symptoms of anxiety and depression in family members of intensive care unit patients: ethical hypothesis regarding decision-making capacity. Crit Care Med. 2001;29(10):1893-1897. doi:10.1097/00003246-200110000-00007 11588447

[19] Azoulay E, Pochard F, Kentish-Barnes N, ; FAMIREA Study Group Risk of post-traumatic stress symptoms in family members of intensive care unit patients. Am J Respir Crit Care Med. 2005;171(9):987-994. doi:10.1164/rccm.200409-1295OC 15665319

[20] Glick DR, Motta M, Wiegand DL, Anticipatory grief and impaired problem solving among surrogate decision makers of critically ill patients: a cross-sectional study. Intensive Crit Care Nurs. 2018;49(July):1-5. doi:10.1016/j.iccn.2018.07.006 30057337

[21] Paulus MP, Yu AJ Emotion and decision-making: affect-driven belief systems in anxiety and depression. Trends Cogn Sci. 2012;16(9):476-483. doi:10.1016/j.tics.2012.07.009 22898207 PMC3446252

[22] Bahadori K, FitzGerald JM Risk factors of hospitalization and readmission of patients with COPD exacerbation–systematic review. Int J Chron Obstruct Pulmon Dis. 2007;2(3):241-251. 18229562 PMC2695199

[23] Quintana JM, Esteban C, Unzurrunzaga A, ; IRYSS-COPD Group Prognostic severity scores for patients with COPD exacerbations attending emergency departments. Int J Tuberc Lung Dis. 2014;18(12):1415-1420. doi:10.5588/ijtld.14.0312 25517805

[24] Mortensen K, Hughes TL Comparing Amazon’s Mechanical Turk platform to conventional data collection methods in the health and medical research literature. J Gen Intern Med. 2018;33(4):533-538. doi:10.1007/s11606-017-4246-0 29302882 PMC5880761

[25] Brown SM, Bell SK, Roche SD, Preferences of current and potential patients and family members regarding implementation of electronic communication portals in intensive care units. Ann Am Thorac Soc. 2016;13(3):391-400. doi:10.1513/AnnalsATS.201509-638OC 26700656

[26] Bell SK, Roche SD, Mueller A, Speaking up about care concerns in the ICU: patient and family experiences, attitudes and perceived barriers. BMJ Qual Saf. 2018;27(11):928-936. doi:10.1136/bmjqs-2017-007525 30002146 PMC6225795

[27] Vasher ST, Zaeh SE, Eakin MN, Turnbull AE Responses to a daughter’s question about prognosis when the patient is expected to die: a qualitative analysis. Ann Am Thorac Soc. 2019;16(12):1595-1598. doi:10.1513/AnnalsATS.201906-435RL 31419913 PMC6956831

[28] Turnbull AE, Hayes MM, Brower RG, Effect of documenting prognosis on the information provided to ICU proxies: a randomized trial. Crit Care Med. 2019;47(6):757-764. doi:10.1097/CCM.0000000000003731 30882479 PMC6897298

[29] Weller JA, Dieckmann NF, Tusler M, Mertz CK, Burns WJ, Peters E Development and testing of an abbreviated numeracy scale: a Rasch analysis approach: Rasch-based numeracy scale. J Behav Decis Making. 2013;26(2):198-212. doi:10.1002/bdm.1751 PMC7161838 32313367

[30] Dugan E, Trachtenberg F, Hall MA Development of abbreviated measures to assess patient trust in a physician, a health insurer, and the medical profession. BMC Health Serv Res. 2005;5(1):64. doi:10.1186/1472-6963-5-64 16202125 PMC1262715

[31] Baker DW, Williams MV, Parker RM, Gazmararian JA, Nurss J Development of a brief test to measure functional health literacy. Patient Educ Couns. 1999;38(1):33-42. doi:10.1016/S0738-3991(98)00116-5 14528569

[32] Chesser AK, Keene Woods N, Wipperman J, Wilson R, Dong F Health literacy assessment of the STOFHLA: paper versus electronic administration continuation study. Health Educ Behav. 2014;41(1):19-24. doi:10.1177/1090198113477422 23444322

[33] Wolfe J, Klar N, Grier HE, Understanding of prognosis among parents of children who died of cancer: impact on treatment goals and integration of palliative care. JAMA. 2000;284(19):2469-2475. doi:10.1001/jama.284.19.2469 11074776

[34] Zier LS, Burack JH, Micco G, Chipman AK, Frank JA, White DB Surrogate decision makers’ responses to physicians’ predictions of medical futility. Chest. 2009;136(1):110-117. doi:10.1378/chest.08-2753 19318665 PMC2716715

[35] White DB, Carson S, Anderson W, A multicenter study of the causes and consequences of optimistic expectations about prognosis by surrogate decision-makers in ICUs. Crit Care Med. 2019;47(9):1184-1193. doi:10.1097/CCM.0000000000003807 31162200 PMC6697218

[36] Murphy DJ, Burrows D, Santilli S, The influence of the probability of survival on patients’ preferences regarding cardiopulmonary resuscitation. N Engl J Med. 1994;330(8):545-549. doi:10.1056/NEJM199402243300807 8302322

[37] Lloyd CB, Nietert PJ, Silvestri GA Intensive care decision making in the seriously ill and elderly. Crit Care Med. 2004;32(3):649-654. doi:10.1097/01.CCM.0000115636.29294.2F 15090942

[38] Chipperfield JG, Hamm JM, Perry RP, Parker PC, Ruthig JC, Lang FR A healthy dose of realism: the role of optimistic and pessimistic expectations when facing a downward spiral in health. Soc Sci Med. 2019;232:444-452. doi:10.1016/j.socscimed.2018.08.030 30409727

[39] Taylor SE, Armor DA Positive illusions and coping with adversity. J Pers. 1996;64(4):873-898. doi:10.1111/j.1467-6494.1996.tb00947.x 8956516

[40] Hamm JM, Kamin ST, Chipperfield JG, Perry RP, Lang FR The detrimental consequences of overestimating future health in late life. J Gerontol B Psychol Sci Soc Sci. 2019;74(3):373-381. doi:10.1093/geronb/gbx074 28633322 PMC6377036

[41] Hart JL, Pflug E, Madden V, Halpern SD Thinking forward: future-oriented thinking among patients with tobacco-associated thoracic diseases and their surrogates. Am J Respir Crit Care Med. 2016;193(3):321-329. doi:10.1164/rccm.201505-0882OC 26436758 PMC4803058

[42] Back AL, Arnold RM, Baile WF, Tulsky JA, Fryer-Edwards K Approaching difficult communication tasks in oncology. CA Cancer J Clin. 2005;55(3):164-177. doi:10.3322/canjclin.55.3.164 15890639

[43] Iyer AS, Dionne-Odom JN, Ford SM, A formative evaluation of patient and family caregiver perspectives on early palliative care in chronic obstructive pulmonary disease across disease severity. Ann Am Thorac Soc. 2019;16(8):1024-1033. doi:10.1513/AnnalsATS.201902-112OC 31039003 PMC6774751

