# Supplementary material for: Effect of Intensivist Communication in a Simulated Setting on Interpretation of Prognosis Among Family Members of Patients at High Risk of Intensive Care Unit Admission: A Randomized Trial
Source: JAMA Netw Open. 2020 Apr 1;3(4):e201945. doi: 10.1001/jamanetworkopen.2020.1945 (PMC7113731; doi:10.1001/jamanetworkopen.2020.1945)
Supplement: Supplement 1. — Trial Protocol [file jamanetwopen-3-e201945-s001.pdf]

The Effect of Intensivist Communication in a Simulated Setting on Interpretation of Prognosis Among Family Members of Patients at High Risk for ICU Admission: A Randomized Trial

Unique Protocol Identification Number: IRB00204036
National Clinical Trial (NCT) Identified Number: NCT04239209
Principal Investigator: Alison Turnbull
Sponsor: Johns Hopkins University
Grant Title: Gordon and Betty Moore Foundation Early Career Award
Grant Number: 4626
Funded by: NHLBI, Gordon and Betty Moore Foundation
Version Number: v.1.000
15 January 2020

Important

The original protocol for this study was reviewed by the Johns Hopkins IRB-6 and determined on August 8, 2019 to qualify as exempt research under the DHHS regulations (IRB00204036). It is available from the principal investigator upon request.

This revised and reformatted version of the study protocol was created in January, 2020 at the request of the reviewing journal.

Table of Contents

Statement of Compliance ... 4
Investigator's Signature ... 4
1 Protocol Summary ... 5
1.1 Synopsis ... 5
1.2 Schema ... 7
1.3 Schedule of Activities ... 8
2 Introduction ... 9
2.1 Study Rationale ... 9
2.2 Background ... 10
2.3 Risk/Benefit Assessment ... 10
2.3.1 Known Potential Risks ... 10
2.3.2 Known Potential Benefits ... 10
2.3.3 Assessment of Potential Risks and Benefits ... 10
3 Objectives and Endpoints ... 11
4 Study Design ... 12
4.1 Overall Design ... 12
4.2 Scientific Rationale for Study Design ... 12
4.3 Justification for Intervention ... 12
4.4 End-of-Study Definition ... 13
5 Study Population ... 14
5.1 Inclusion Criteria ... 14
5.2 Exclusion Criteria ... 14
5.3 Lifestyle Considerations ... 14
5.4 Screen Failures ... 14
5.5 Strategies for Recruitment and Retention ... 14
6 Study Intervention(s) or Experimental Manipulation(s) ... 15
6.1 Study Intervention(s) or Experimental Manipulation(s) Administration ... 15
6.1.1 Study Intervention or Experimental Manipulation Description ... 15
6.1.2 Administration and/or Dosing ... 15
6.2 Fidelity ... 15
6.2.1 INTERVENTIONIST TRAINING AND TRACKING ... 15
6.3 Measures to Minimize Bias: Randomization and Blinding ... 15
6.4 Study Intervention/Experimental Manipulation Adherence ... 15
6.5 Concomitant Therapy ... 15
6.5.1 Rescue Therapy ... 16
7 Study Intervention/Experimental Manipulation Discontinuation and Participant Discontinuation/Withdrawal ... 17
7.1 Discontinuation of Study Intervention/Experimental Manipulation ... 17
7.2 Participant Discontinuation/Withdrawal from the Study ... 17
7.3 Lost to Follow-Up ... 17
8 Study Assessments and Procedures ... 18
8.1 Endpoint and Other Non-Safety Assessments ... 18
8.2 Safety Assessments ... 19
8.3 Adverse Events and Serious Adverse Events ... 19
8.4 Unanticipated Problems ... 19
8.4.1Definition of Unanticipated Problems ... 19
8.4.2Unanticipated Problems Reporting ... 20

8.4.3 Reporting Unanticipated Problems to Participants 20
9 Statistical Considerations 21
9.1 Statistical Hypotheses 21
9.2 Sample Size Determination 21
9.3 Populations for Analyses 21
9.4 Statistical Analyses 21
9.4.1 General Approach 21
9.4.2 Analysis of the Primary Endpoint(s) 22
9.4.3 Analysis of the Secondary Endpoint(s) 22
9.4.4 Safety Analyses 22
9.4.5 Baseline Descriptive Statistics 22
9.4.6 Planned Interim Analyses 22
9.4.7 Sub-Group Analyses 22
9.4.8 Tabulation of Individual Participant Data 22
9.4.9 Exploratory Analyses 22
10 Supporting Documentation and Operational Considerations 24
10.1 Regulatory, Ethical, and Study Oversight Considerations 24
10.1.2Study Discontinuation and Closure 24
10.1.3Confidentiality and privacy 24
10.1.4Future Use of Stored Specimens and Data 25
10.1.6Safety Oversight 25
10.1.7Clinical Monitoring 25
10.1.8Quality Assurance and Quality Control 25
10.1.9Data Handling and Record Keeping 25
10.1.10 Protocol Deviations 26
10.1.11 Publication and Data Sharing Policy 26
10.1.12 Conflict of Interest Policy 26
10.2 Additional Considerations 26
10.3 Abbreviations and Special Terms 26
10.4 Protocol Amendment History 26
11 References 28
References 28

STATEMENT OF COMPLIANCE

All personnel involved in the conduct of this study have completed Human Subjects Protection and ICH GCP Training.

The protocol, informed consent form(s), and all participant materials will be submitted to the IRB for review and approval. Approval of both the protocol and the consent form(s) must be obtained before any participant is consented. Any amendment to the protocol will require review and approval by the IRB before the changes are implemented to the study. All changes to the consent form(s) will be IRB approved.

INVESTIGATOR'S SIGNATURE

Principal Investigator or Clinical Site Investigator:

Signed: Date: 1/21/2020

Name*: Alison E. Turnbull

Title*: Assistant Professor

Investigator Contact Information:

Affiliation*: Johns Hopkins University
Address: 1830 E. Monument St
Telephone: 410-955-2190
Email: turnbull@jhmi.edu

1 PROTOCOL SUMMARY

1.1 SYNOPSIS

Title: The Effect of Intensivist Communication on Prognosis Interpretation by Family Members of Patients at High Risk for Intensive Care Unit Admission: A Randomized Trial

Grant Number: 4626

Study Description: This study evaluates the effect of physician communication styles on the interpretation of prognosis by family members of chronically-ill patients. Participants were randomized to view one of four videos how depicting different physicians disclose prognosis when they expect an ICU patient to die.

Objectives*:

Primary Objective:	Quantify participant perceptions of the intensivist's prognostic estimate.
Secondary Objectives:	1) Quantify participant prognostic estimates.<br>2) Quantify participant differences in belief about prognosis.<br>3) Describe participant confidence that they understood the intensivist's belief about prognosis.<br>4) Describe participant confidence in their own prognostic estimate.

Endpoints*:

Primary Endpoint:	Participant response to the question "If you had to guess, what do you think the doctor thinks is the chance that your loved one will survive this hospitalization?” answered on a 0-100% probability scale.
Secondary Endpoints:	1) Participant response to the question "What do you think are the chances that your loved one will survive this hospitalization?" answered on a 0-100% probability scale.<br>2) The difference between the participant's prognostic estimate and the participant's perception of the intensivist's prognostic estimate.<br>3) Participant confidence in their ability to interpret the doctor's prognostic estimate of survival (primary outcome) using a 5-item Likert scale.<br>4) Participant confidence in their own estimate of their loved one's chances of survival to discharge using a 5-item Likert scale.

Study Population: People age ≥18 years who are a spouse/partner, sibling, or adult child of a patient with Chronic Obstructive Pulmonary Disease (COPD) on home oxygen.

Phase* or Stage: n/a

Description of Sites/Facilities Enrolling Participants: Web-based trial.

**Description of Study Intervention/Experimental Manipulation:**	One of four videos depicting different intensivist communication styles.
**Study Duration*:**	3 weeks
**Participant Duration:**	20 minutes on average

1.2 SCHEMA

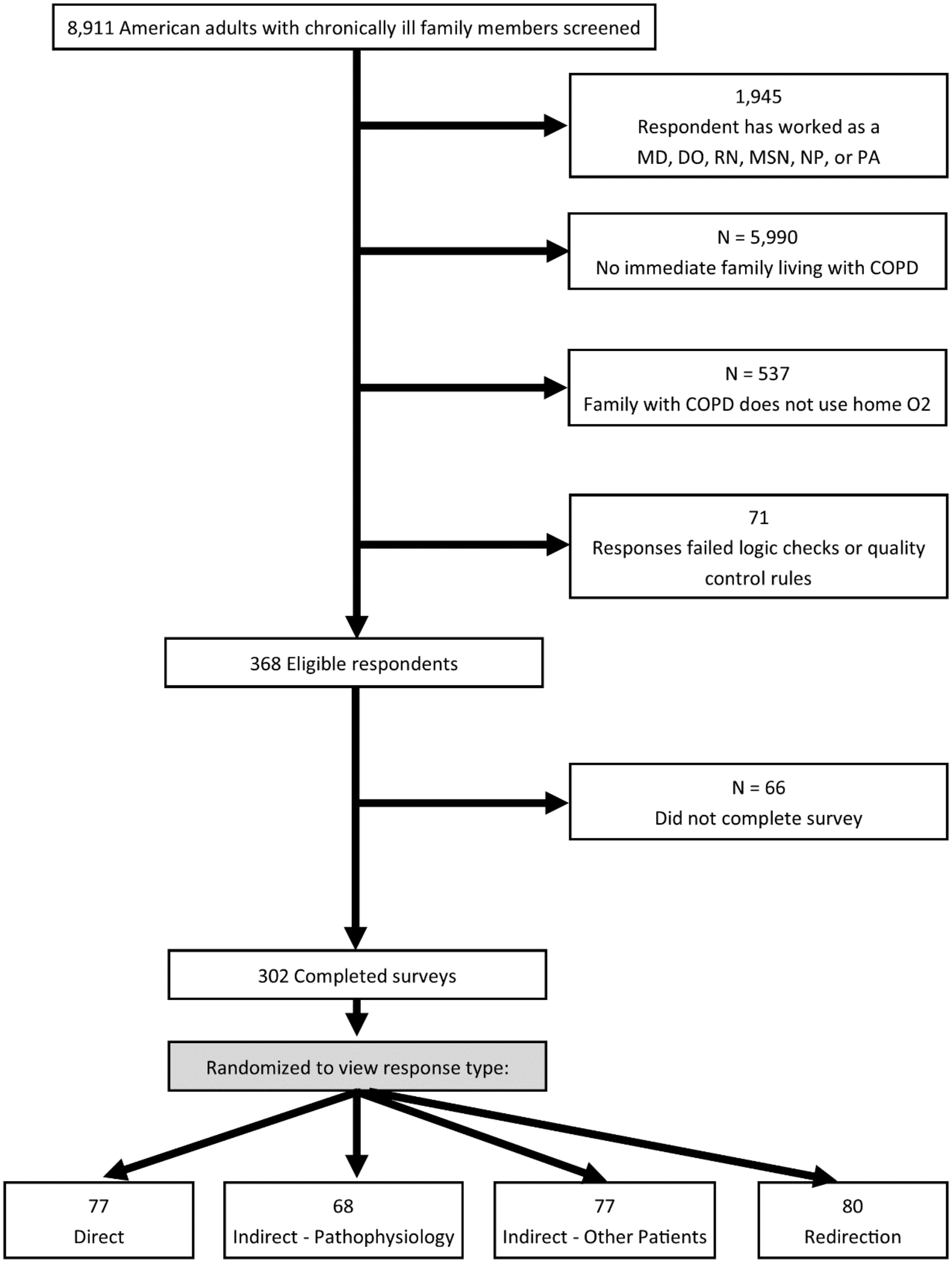
8,911 American adults with chronically ill family members screened
1,945
Respondent has worked as a
MD, DO, RN, MSN, NP, or PA
N = 5,990
No immediate family living with COPD
N = 537
Family with COPD does not use home O2
71
Responses failed logic checks or quality
control rules
368 Eligible respondents
N = 66
Did not complete survey
302 Completed surveys
Randomized to view response type:
77
Direct
68
Indirect - Pathophysiology
77
Indirect - Other Patients
80
Redirection

1.3 SCHEDULE OF ACTIVITIES

This is an online-only randomized trial. When the participant consents to participate the trial begins. Once all study questions are answered the participant's involvement in the study ceases.

2 INTRODUCTION

2.1 STUDY RATIONALE

The majority of Americans surveyed >65 years old would prefer to forego mechanical ventilation and other life support therapies and, instead, die at home with supportive care[1,2] and most patients do not change their end of life preferences over time or after changes in health status.[3] **However, Intensive Care Unit (ICU) use during the last month of life has increased for >15 years to nearly 30%.**[4,5,6] Additionally, among hospitalized patients with advanced dementia and severe functional impairment, **mechanical ventilation use doubled between 2000-2013 without a significant improvement in survival**.[7] Compared to other developed countries, the United States has a much higher use of intensive care services at the end of life.[8,9] Death in an ICU amongst patients with cancer has been associated with more physical and emotional distress and worse quality of life at the end of life, in addition to increased risk of psychiatric illness in bereaved caregivers compared to those who die at home with hospice.[10] This discordance between preferred and actual site of death, led to the following recommendation by the American Board of internal Medicine Foundation's *Choosing Wisely* campaign: ***"Don't continue life support for patients at high risk for death or severely impaired functional recovery without offering patients and their families the alternative of care focused entirely on comfort."***[11] This study will help clarify how to clearly convey the prognosis of ICU patients, which is essential to do before offering care focused on comfort.

Studying Communication in the ICU: Because critical illness prevents many patients from communicating with the medical team, family surrogates frequently act as decision-makers for most ICU patients.[12] Effective communication about prognosis with these surrogates is essential to ensuring they make informed decisions about their loved one's care.[13] Indeed, the Institute of Medicine's report *Dying in America: Improving Quality and Honoring Individual Preferences Near the End of Life* recommends **improving clinicians' ability to talk effectively to patients about dying.**[14] When the option of speaking directly to the patient is absent, such as in the ICU, this recommendation also applies to patient surrogates. Previous trials have examined different methods for communicating prognosis to ICU surrogates,[15 16] but **substantial intensivist-surrogate discordance about prognosis remains** [15 17 18] even when surrogates rate the quality of physician communication highly.[19] Recent research suggests intensivist-surrogate discordance about prognosis is a result of both surrogate misunderstanding and differences between physician and surrogate belief systems.[17 20] While surrogate belief systems are largely immutable, **misunderstandings due to communication failures are a correctable target for intervention.**

Limitations in Current Research Methods: Most research on communicating with ICU surrogates has studied surrogates of current ICU patients. Anxiety, depression, sleep deprivation, anticipatory grief, and post-traumatic stress disorder are common in this population,[21 22 23 24] and may affect decision making.[21 22 25] Surveys aimed at surrogates of current ICU patients may be biased towards respondents who are white,[26] visit the hospital more frequently,[27] and are of a higher socioeconomic status.[26 28] To address these problems, we will study close family members (spouses, siblings, and adult children) of outpatients who are ill enough that ICU admission in the near future is likely, thus examining a population of potential surrogates who are not acutely stressed about a loved one's day-to-day survival in the ICU. Specifically, we will study family members of adults with Chronic Obstructive Pulmonary Disease (COPD) requiring home oxygen, as these family members are likely to become patient surrogates in the near future.[29 30]

2.2 BACKGROUND

Foundational studies leading to this current protocol included a vignette-based online randomized trial which demonstrated that requiring intensivists to record patients' expected functional prognosis substantially increased their intention to discuss withdrawing life support.[31] A subsequent double-blind randomized controlled trial was then performed to test the effect of requiring intensivists to document short-term and long-term functional prognosis on their communication behaviors in a family meeting conducted with an actor in a well-controlled, high-fidelity, hospital-based simulation center (ClinicalTrials.gov NCT02721810).[32] A total of 116 U.S. intensivists were recruited to the Johns Hopkins Hospital Simulation Center where they reviewed paper-based medical records of a hypothetical patient scenario, developed so that in-hospital death was highly probable but not certain. Physicians in both the control and intervention groups were then asked a series of questions about their management plan; intensivists randomized to the intervention group also answered 3 additional questions about the hypothetical patient's prognosis. One question, which we will use the responses to in our study, was "Do you expect this patient to survive to hospital discharge?"[15 17] All intensivists then participated in a standardized, video- and audio-recorded, simulated family meeting, using specially trained and paid actors portraying the patient's daughter. Two blinded ICU-physician reviewers then analyzed each transcribed recording. Study participant behaviors and language during the simulations then underwent a qualitative analysis and categorization into different communication patterns by two blinded physician reviewers. The analysis centered on the response of the intensivist to a portion of the simulation where the actor was told to signpost a specific desire to know prognosis by asking the question "*What do you think is most likely to happen*?"[33] From this analysis, four videos were selected to demonstrate archetypal physician communication patterns - direct communication, indirect focusing on physiology, indirect focusing on other patients, and redirection. Each video came from an intensivist who thought that the hypothetical patient would not survive the hospital stay, and each intensivist answered that they had conveyed the prognosis for a risk of death to the family member in the simulation.

2.3 RISK/BENEFIT ASSESSMENT

2.3.1 KNOWN POTENTIAL RISKS

There is a small risk of loss of confidentiality.

2.3.2 KNOWN POTENTIAL BENEFITS

Participants will not individually benefit from this research. The proposed research has the potential to support guidelines and interventions to improve prognosis communication in the intensive care unit.

2.3.3 ASSESSMENT OF POTENTIAL RISKS AND BENEFITS

The risks involved with this study are minimal and benefits to ICU care in general could be large.

3 OBJECTIVES AND ENDPOINTS

OBJECTIVES	ENDPOINTS	JUSTIFICATION FOR ENDPOINTS	PUTATIVE MECHANISMS OF ACTION
Primary			
Quantify participant perceptions of the intensivist's prognostic estimate	Participant response to the question "If you had to guess, what do you think the doctor thinks is the chance that your loved one will survive this hospitalization?" answered on a 0-100% probability scale.	This exact question has been used in a prior landmark study of of prognosis communication with ICU proxies.[17]	Health literacy, health numeracy, physician communication skills and word choice.
Secondary			
Quantify participant prognostic estimates.	Participant response to the question "What do you think are the chances that your loved one will survive this hospitalization?" answered on a 0-100% probability scale.	This exact question has been used in a prior landmark study of of prognosis communication with ICU proxies.[17]	Optimism, hope, religious faith, trust in physicians, misunderstanding, health literacy, health numeracy, physician communication skills and word choice.
Quantify participant difference in belief about prognosis.	The difference between the participant's prognostic estimate and the participant's perception of the intensivist's prognostic estimate.	This definition was established in a prior landmark study on prognosis communication with ICU proxies.[17]	Optimism, hope, religious faith, trust in physicians
Tertiary/Exploratory			
Describe participant confidence that they understood the intensivist's belief about prognosis.	Participant confidence in their ability to interpret the doctor's prognostic estimate of survival (primary outcome) using a 5-item Likert scale.	Exploratory outcome.	Prior experience speaking with intensivists.
Describe participant confidence in their own prognostic estimate.	Participant confidence in their own estimate of their loved one's chances of survival to discharge using a 5-item Likert scale.	Exploratory outcome.	Prior experience advocating for a critically ill loved one.

4 STUDY DESIGN

4.1 OVERALL DESIGN

Study Type: Interventional
Primary Purpose: Other
Study Phase: N/A
Interventional Study Model: Parallel Assignment
Participants are randomized in a 1:1:1:1 ratio to view a video depicting one of four different ways intensivists answered a patient surrogate's prognostic question "What do you think is most likely to happen?" during a simulated ICU family meeting.
Number of Arms: 4
Masking: None (Open Label)
Allocation: Randomized
Enrollment: 302 [Actual]

4.2 SCIENTIFIC RATIONALE FOR STUDY DESIGN

A randomized trial is the ideal way to study the effect of different intensivist communication styles on surrogate prognosis interpretation. We have designed our trial to be simulation-based in order to avoid the ethical dilemma of randomly subjecting surrogates of current ICU patients to experiencing different communication styles when intensivists deliver prognosis, some of which we hypothesize to be less effective. We have also designed the trial to recruit a sample of people who are not currently acting as surrogates for their family member in an ICU but have a high likely of doing so in the future. This trial design also allows us to control for the many factors which are hypothesized to affect how families interpret statements about prognosis in the ICU.

4.3 JUSTIFICATION FOR INTERVENTION

Previous research has shown that intensivists convey prognosis in many different ways. The interventions, videos depicting different styles of communicating prognosis, were created from verbatim transcripts obtained during a prior simulation trial (see 2.2 Background). A prior qualitative study (see 2.2 Background) then classified the videos into four categories of communication style, which are used in the present trial as an intervention.

4.4 END-OF-STUDY DEFINITION

Sample size has been accrued and all participants have finished the single online trial session.

5 STUDY POPULATION

5.1 INCLUSION CRITERIA

Spouse/partner, sibling, or adult child of a patient with Chronic Obstructive Pulmonary Disease (COPD) on home oxygen, and ≥ 18 years old.

5.2 EXCLUSION CRITERIA

Current or previous experience as a nurse, advanced practice provider, or physician

5.3 LIFESTYLE CONSIDERATIONS

N/A

5.4 SCREEN FAILURES

Screen failures will be excluded from the study and partial responses will not be analyzed.

5.5 STRATEGIES FOR RECRUITMENT AND RETENTION

Recruitment will occur via Qualtrics LLC, who will distribute our online trial to their proprietary pre-existing survey panels.

6 STUDY INTERVENTION(S) OR EXPERIMENTAL MANIPULATION(S)

6.1 STUDY INTERVENTION(S) OR EXPERIMENTAL MANIPULATION(S) ADMINISTRATION

6.1.1 STUDY INTERVENTION OR EXPERIMENTAL MANIPULATION DESCRIPTION

The intervention consists of a short (< 1 minute) video of an intensive care physician communicating a hypothetical patient's prognosis directly to the camera. The trial participant will be asked to imagine that their loved one with COPD is in the ICU, that they have asked the doctor the question "What do you think is most likely to happen?" and that the video depicts their doctor's response to that question.

6.1.2 ADMINISTRATION AND/OR DOSING

This is a web-based video hosted on a video streaming website. It is integrated into the Qualtrics online platform.

6.2 FIDELITY

6.2.1 INTERVENTIONIST TRAINING AND TRACKING

N/A

6.3 MEASURES TO MINIMIZE BIAS: RANDOMIZATION AND BLINDING

Randomization occurs via a computer algorithm integrated within the Qualtrics online platform. Participants will be randomized in a 1:1:1:1 ratio to view one of the four study videos.

6.4 STUDY INTERVENTION/EXPERIMENTAL MANIPULATION ADHERENCE

N/A - the study consists of only one online encounter.

6.5 CONCOMITANT THERAPY

6.5.1 RESCUE THERAPY

N/A

7 STUDY INTERVENTION/EXPERIMENTAL MANIPULATION DISCONTINUATION AND PARTICIPANT DISCONTINUATION/WITHDRAWAL

7.1 DISCONTINUATION OF STUDY INTERVENTION/EXPERIMENTAL MANIPULATION

N/A

7.2 PARTICIPANT DISCONTINUATION/WITHDRAWAL FROM THE STUDY

Participants may withdraw from answering study questions at any time by closing their web browser.

7.3 LOST TO FOLLOW-UP

N/A - There is only one study session as part of this online trial.

8 STUDY ASSESSMENTS AND PROCEDURES

8.1 ENDPOINT AND OTHER NON-SAFETY ASSESSMENTS

The entire trial occurs online. Participants are asked the following questions:

Screening questions:
Have you ever worked as a doctor, nurse, or advanced practice provider (PA, NP, MSN, etc.)? [Yes, No]
How old are you?
Are you the spouse, partner, child, or sibling of someone with any of the following conditions? [renal (kidney) disease requiring regular dialysis, Chronic Obstructive Pulmonary Disease (also called COPD or emphysema, Severe dementia, None of the above]
Is your loved one with Chronic Obstructive Pulmonary Disease (also called COPD or emphysema) deceased? [Yes, No]
Does your loved one with Chronic Obstructive Pulmonary Disease (also called COPD or emphysema) require oxygen at home? [Yes, No]
Has your loved one with COPD ever needed a breathing machine (also known as a ventilator) during a hospital stay? [Yes, No, Unsure/I don't know]
What best describes your relationship to the person with Chronic Obstructive Pulmonary Disease (also called COPD or emphysema) requiring oxygen at home? [Spouse/Partner, Parent/Step-Parent, Sibling, None of the above]

Participants are then given the simulation instructions and instructions for watching the video.

The video then plays.

Study questions are asked immediately after the video is complete:
What do you hope (best case scenario) is the chance that your loved one survives the hospitalization? [answered on a 0-100% scale]
What do you think are the chances that your loved one will survive this hospitalization? [answered on a 0-100% scale]
How confident are you that you understand your loved one's chances for surviving the hospitalization? [answered on a 5-item Likert scale]
If you had to guess, what do you think the doctor thinks is the chance that your loved one will survive this hospitalization? [answered on a 0-100% scale]
How confident are you that you know what the doctor thinks your loved one's chances for surviving the hospitalization are? [answered on a 5-item Likert scale]

Standardized assessments are then administered:
Wake Forest Physician Trust Scale
Demographic questions [education level, ethnicity, race, sex, ZIP code]

Participants then view 3 of 16 hypothetical, previously-validated text-based prognostic statements and are then asked to answer their prognostic interpretation on a 0-100% scale.

Two more standardized assessments are then administered:
Short Test of Functional Health Literacy in Adults
Rasch-based numeracy assessment

Finally, the survey ends. An option appears for two further optional questions which the participant may answer if they choose to. Completion of these two questions is not considered criteria for study completion:
Optional Free text box allowing the participant to write any comments they have about how doctors and family members of sick patients communicate.
Optional area to enter name, telephone, and or email to be contacted about future studies.

8.2 SAFETY ASSESSMENTS

N/A

8.3 ADVERSE EVENTS AND SERIOUS ADVERSE EVENTS

N/A

8.4 UNANTICIPATED PROBLEMS

8.4.1 DEFINITION OF UNANTICIPATED PROBLEMS

This protocol uses the definition of Unanticipated Problems as defined by the Office for Human Research Protections (OHRP). OHRP considers unanticipated problems involving risks to participants or others to include, in general, any incident, experience, or outcome that meets **all** of the following criteria:

- Unexpected in terms of nature, severity, or frequency given (a) the research procedures that are described in the protocol-related documents, such as the Institutional Review Board (IRB)-approved research protocol and informed consent document; and (b) the characteristics of the participant population being studied;
- Related or possibly related to participation in the research ("possibly related" means there is a reasonable possibility that the incident, experience, or outcome may have been caused by the procedures involved in the research); and
- Suggests that the research places participants or others at a greater risk of harm (including physical, psychological, economic, or social harm) than was previously known or recognized.

8.4.2 UNANTICIPATED PROBLEMS REPORTING

The investigator will report unanticipated problems (UPs) to the reviewing Institutional Review Board (IRB) and to the lead principal investigator (PI). The UP report will include the following information:

- Protocol identifying information: protocol title and number, PI's name, and the IRB project number
- A detailed description of the event, incident, experience, or outcome
- An explanation of the basis for determining that the event, incident, experience, or outcome represents an UP
- A description of any changes to the protocol or other corrective actions that have been taken or are proposed in response to the UP

To satisfy the requirement for prompt reporting, UPs will be reported using the following timeline:

- UPs that are serious adverse events (SAEs) will be reported to the IRB within 7 business days of the investigator becoming aware of the event
- Any other UP will be reported to the IRB within 7 business days of the investigator becoming aware of the problem
- All UPs should be reported to appropriate institutional officials (as required by an institution's written reporting procedures), the supporting agency head (or designee), and the Office for Human Research Protections (OHRP) within 7 business days of the IRB's receipt of the report of the problem from the investigator

8.4.3 REPORTING UNANTICIPATED PROBLEMS TO PARTICIPANTS

Our trial protocol does not include collection of personally identifiable information, except for an optional section in which the participant can leave an email address or phone number to be contacted about future studies. If necessary to report unanticipated problems to participants we can report to only those who have optionally provided their contact information.

9 STATISTICAL CONSIDERATIONS

9.1 STATISTICAL HYPOTHESES

- Primary Efficacy Endpoint(s):

We hypothesize that participants will perceive intensivists to be less optimistic when randomized to view a direct answer to their question about prognosis.

- Secondary Efficacy Endpoint(s):

We hypothesize that participants will be less optimistic when they are randomized to view a direct answer to their question about prognosis.

We hypothesize that differences in belief about prognosis (defined as the difference between the proxy's prognostic estimate and the intensivist's perceived estimate) will be unaffected by intensivist communication style.

9.2 SAMPLE SIZE DETERMINATION

Based on surrogate decision makers' interpretations of prognostic information in previous research we estimated that enrolling 75 participants in each arm of the trial would provide power of 0.9 to detect at least an 8 point difference in the mean interpretation of intensivist statements assuming a 2-sided α of 0.05, and an estimated standard deviation of 15.

9.3 POPULATIONS FOR ANALYSES

The per-protocol population will be analyzed. Participants who did not complete the trial will not be analyzed.

9.4 STATISTICAL ANALYSES

9.4.1 GENERAL APPROACH

Descriptive statistics and data visualizations will be used to summarize primary and secondary endpoints, baseline descriptive statistics, and exploratory analyses as described below. Generalized linear regression will be used to estimate the effect of intensivist communication style on primary and secondary outcomes. All analyses will assume a 2-sided alpha of 0.05.

9.4.2 ANALYSIS OF THE PRIMARY ENDPOINT(S)

Multivariable linear regression will be used to estimate the effect of communication pattern (i.e. which video the participant is randomized to view) on perception of the intensivist's prognostic estimate. The direct communication pattern will be treated as the comparator (control group) in all models. All models will be adjusted for the following variables which are hypothesized to confound the relationship between physician communication style and proxy perception of the intensivist's prognostic estimate: 1) the relationship of the participant to their loved one, 2) whether the participant’s loved one has previously been mechanically ventilated, 3) education level, 4) numeracy score, 5) trust in physicians score, and 6) health literacy score. Residual plots will be reviewed to evaluate model assumptions. Unadjusted models will also be reported.

9.4.3 ANALYSIS OF THE SECONDARY ENDPOINT(S)

Multivariable linear regression will be used to estimate the effect of communication pattern (i.e. which video the participant is randomized to view) on the participant's prognostic estimate and participant difference in belief. The direct communication pattern will be treated as the comparator (control group) in all models. All models will be adjusted for the following variables which are hypothesized to confound the relationship between physician communication style and participant's prognostic estimate and difference in belief: 1) the relationship of the participant to their loved one, 2) whether the participant’s loved one has previously been mechanically ventilated, 3) education level, 4) numeracy score, 5) trust in physicians score, and 6) health literacy score. Residual plots will be reviewed to evaluate model assumptions. Unadjusted models will also be reported.

9.4.4 SAFETY ANALYSES

N/A

9.4.5 BASELINE DESCRIPTIVE STATISTICS

Descriptive statistics, either median value with interquartile range or counts and percentages, will be reported for demographic questions as well as participant relationship to the patient, prior experience advocating for someone receiving mechanical ventilation, numeracy score, Wake Forest physician trust scale, and health literacy score.

9.4.6 PLANNED INTERIM ANALYSES

N/A

9.4.7 SUB-GROUP ANALYSES

No specific sub-group analyses are planned.

9.4.8 TABULATION OF INDIVIDUAL PARTICIPANT DATA

N/A

9.4.9 EXPLORATORY ANALYSES

Participants who selected “Confident” or “Very confident” on the 5-point Likert scale are analyzed as being confident with a sensitivity analysis performed to ensure estimates are robust to the threshold chosen for confidence (e.g. including "neutral" as a confident response). The difference in proportion of confident participants is estimated using the sample proportions in each trial arm compared to the control group. The null hypothesis of no difference is tested using the Fisher exact test.

10 SUPPORTING DOCUMENTATION AND OPERATIONAL CONSIDERATIONS

10.1 REGULATORY, ETHICAL, AND STUDY OVERSIGHT CONSIDERATIONS

10.1.1.1 CONSENT/ASSENT AND OTHER INFORMATIONAL DOCUMENTS PROVIDED TO PARTICIPANTS

The following language will be presented at the beginning of the online trial:

"You have been selected as a potential participant in a survey about how doctors communicate. This survey is anonymous and no identifying information will be collected. There are no right or wrong answers. The survey should take about 15 minutes to complete and will involve watching a short video clip of a doctor answering a question. If you are upset by the video or survey you can stop at any time. If you take part in this study, you may help others in the future by helping doctors and family members of patients work together in healthcare. Selected information from this survey might be made available for other researchers to work with through a publicly-available database, but no identifying information will be collected by this survey or uploaded to this database. If you have questions about this survey please contact the study team representative Ian Oppenheim at ian@jhmi.edu or the principal investigator Alison Turnbull at turnbull@jhmi.edu. Clicking "I Agree" below will begin the survey and serve as your consent to participate. If you do not agree, please close this web page."

10.1.2 STUDY DISCONTINUATION AND CLOSURE

This study may be temporarily suspended or prematurely terminated if there is sufficient reasonable cause. Circumstances that may warrant termination or suspension include, but are not limited to:

- Determination of unexpected, significant, or unacceptable risk to participants
- Data that are not sufficiently complete and/or evaluable

10.1.3 CONFIDENTIALITY AND PRIVACY

Participant confidentiality and privacy is strictly held in trust by the participating investigators, their staff, the safety and oversight monitor(s), and the sponsor(s) and funding agency. This confidentiality is extended to the data being collected as part of this study. Data that could be used to identify a specific study participant will be held in strict confidence within the research team. No personally-identifiable information from the study will be released to any unauthorized third party without prior written approval of the sponsor/funding agency.

The study monitor, other authorized representatives of the sponsor, representatives of the Institutional Review Board (IRB), or regulatory agencies may inspect all documents and records required to be maintained by the investigator. The clinical study site will permit access to such records. At the end of the study, all records will continue to be kept in a secure location for as long a period as dictated by the reviewing IRB, Institutional policies, or sponsor requirements.

Study participant research data, which is for purposes of statistical analysis and scientific reporting, will be transmitted to and stored at the Johns Hopkins University. This will not include the participant's contact or identifying information. Rather, individual participants and their research data will be identified by a unique study identification number. The study data entry and study management systems used by study site research staff will be secured and password protected. At the end of the study, all study databases will be de-identified and archived at the Johns Hopkins University.

The PI will ensure all mechanisms used to share data will include proper plans and safeguards for the protection of privacy, confidentiality, and security for data dissemination and reuse (e.g., all data will be thoroughly de-identified and will not be traceable to a specific study participant). Plans for archiving and long-term preservation of the data will be implemented, as appropriate.

10.1.4 FUTURE USE OF STORED SPECIMENS AND DATA

Data collected for this study will be analyzed and stored at the Johns Hopkins University. After the study is completed, the de-identified, archived data will be transmitted to and stored at the Johns Hopkins University Data Archive. When the study is completed, access to archived data will be provided through Johns Hopkins University.

10.1.6 SAFETY OVERSIGHT

N/A

10.1.7 CLINICAL MONITORING

N/A

10.1.8 QUALITY ASSURANCE AND QUALITY CONTROL

N/A

10.1.9 DATA HANDLING AND RECORD KEEPING

10.1.9.1 DATA COLLECTION AND MANAGEMENT RESPONSIBILITIES

Data collection will be the responsibility of the clinical trial staff at the site under the supervision of the site investigator. The investigator will be responsible for ensuring the accuracy, completeness, legibility, and timeliness of the data reported.

10.1.9.2 STUDY RECORDS RETENTION

Study documents will be retained for a minimum of 2 years after formal discontinuation of the study. It is the responsibility of the investigator to determine when these documents no longer need to be retained.

10.1.10 PROTOCOL DEVIATIONS

This protocol defines a protocol deviation as any noncompliance with the clinical trial protocol. The noncompliance may be either on the part of the participant, the investigator, or the study site staff. As a result of deviations, corrective actions will be developed by the site and implemented promptly.

10.1.11 PUBLICATION AND DATA SHARING POLICY

This study will be conducted in accordance with the following publication and data sharing policies and regulations:
National Institutes of Health (NIH) Public Access Policy, which ensures that the public has access to the published results of NIH funded research. It requires scientists to submit final peer-reviewed journal manuscripts that arise from NIH funds to the digital archive PubMed Central upon acceptance for publication.
This study will comply with the NIH Data Sharing Policy and Policy on the Dissemination of NIH-Funded Clinical Trial Information and the Clinical Trials Registration and Results Information Submission rule. As such, this trial will be registered at ClinicalTrials.gov, and results information from this trial will be submitted to ClinicalTrials.gov. In addition, every attempt will be made to publish results in peer-reviewed journals. Considerations for ensuring confidentiality of these shared data are described in Section 10.1.3.

10.1.12 CONFLICT OF INTEREST POLICY

The independence of this study from any actual or perceived influence, such as by the pharmaceutical industry, is critical. Therefore any actual conflict of interest of persons who have a role in the design, conduct, analysis, publication, or any aspect of this trial will be disclosed and managed. Furthermore, persons who have a perceived conflict of interest will be required to have such conflicts managed in a way that is appropriate to their participation in the design and conduct of this trial.

10.2 ADDITIONAL CONSIDERATIONS

10.3 ABBREVIATIONS AND SPECIAL TERMS

10.4 PROTOCOL AMENDMENT HISTORY

Version	Date	Description of Change	Brief Rationale

11 REFERENCES

REFERENCES

1. Fischer S, Min SJ, Cervantes L, Kutner J, Where do you want to spend your last days of life? Low concordance between preferred and actual site of death among hospitalized adults., J Hosp Med. 2013 Apr;8(4):178-83

   Pmid:23440934

2. Barnato AE, Herndon MB, Anthony DL, Gallagher PM, Skinner JS, Bynum JP, Fisher ES, Are regional variations in end-of-life care intensity explained by patient preferences?: A Study of the US Medicare Population., Med Care. 2007 May;45(5):386-93

   Pmid:17446824

3. Auriemma CL, Nguyen CA, Bronheim R, Kent S, Nadiger S, Pardo D, Halpern SD, Stability of end-of-life preferences: a systematic review of the evidence., JAMA Intern Med. 2014 Jul;174(7):1085-92

   Pmid:24861560

4. Needham DM, Bronskill SE, Calinawan JR, Sibbald WJ, Pronovost PJ, Laupacis A, Projected incidence of mechanical ventilation in Ontario to 2026: Preparing for the aging baby boomers., Crit Care Med. 2005 Mar;33(3):574-9

   Pmid:15753749

5. Angus DC, Barnato AE, Linde-Zwirble WT, Weissfeld LA, Watson RS, Rickert T, Rubenfeld GD, Robert Wood Johnson Foundation ICU End-Of-Life Peer Group., Use of intensive care at the end of life in the United States: an epidemiologic study., Crit Care Med. 2004 Mar;32(3):638-43

   Pmid:15090940

6. Teno JM, Gozalo P, Trivedi AN, Bunker J, Lima J, Ogarek J, Mor V, Site of Death, Place of Care, and Health Care Transitions Among US Medicare Beneficiaries, 2000-2015., JAMA. 2018 Jul 17;320(3):264-271

   Pmid:29946682

7. Teno JM, Gozalo P, Khandelwal N, Curtis JR, Meltzer D, Engelberg R, Mor V, Association of Increasing Use of Mechanical Ventilation Among Nursing Home Residents With Advanced Dementia and Intensive Care Unit Beds., JAMA Intern Med. 2016 Dec 1;176(12):1809-1816

Pmid:27723891

8. Wunsch H, Linde-Zwirble WT, Harrison DA, Barnato AE, Rowan KM, Angus DC, Use of intensive care services during terminal hospitalizations in England and the United States., Am J Respir Crit Care Med. 2009 Nov 1;180(9):875-80

Pmid:19713448

9. Bekelman JE, Halpern SD, Blankart CR, Bynum JP, Cohen J, Fowler R, Kaasa S, Kwietniewski L, Melberg HO, Onwuteaka-Philipsen B, Oosterveld-Vlug M, Pring A, Schreyögg J, Ulrich CM, Verne J, Wunsch H, Emanuel EJ, International Consortium for End-of-Life Research (ICELR)., Comparison of Site of Death, Health Care Utilization, and Hospital Expenditures for Patients Dying With Cancer in 7 Developed Countries., JAMA. 2016 Jan 19;315(3):272-83

Pmid:26784775

10. Wright AA, Keating NL, Balboni TA, Matulonis UA, Block SD, Prigerson HG, Place of death: correlations with quality of life of patients with cancer and predictors of bereaved caregivers' mental health., J Clin Oncol. 2010 Oct 10;28(29):4457-64

Pmid:20837950

11. Halpern SD, Becker D, Curtis JR, Fowler R, Hyzy R, Kaplan LJ, Rawat N, Sessler CN, Wunsch H, Kahn JM, Choosing Wisely Taskforce., American Thoracic Society., American Association of Critical-Care Nurses., Society of Critical Care Medicine., An official American Thoracic Society/American Association of Critical-Care Nurses/American College of Chest Physicians/Society of Critical Care Medicine policy statement: the Choosing Wisely® Top 5 list in Critical Care Medicine., Am J Respir Crit Care Med. 2014 Oct 1;190(7):818-26

Pmid:25271745

12. Silveira MJ, Kim SY, Langa KM, Advance directives and outcomes of surrogate decision making before death., N Engl J Med. 2010 Apr 1;362(13):1211-8

Pmid:20357283

13. Turnbull AE, Hartog CS, Goal-concordant care in the ICU: a conceptual framework for future research., Intensive Care Med. 2017 Dec;43(12):1847-1849

Pmid:28656453

14. Committee on Approaching Death: Addressing Key End of Life Issues, Institute of Medicine, , . 2015 Mar 19;

Pmid:25927121

15. Lee Char SJ, Evans LR, Malvar GL, White DB, A randomized trial of two methods to disclose prognosis to surrogate decision makers in intensive care units., Am J Respir Crit Care Med. 2010 Oct 1;182(7):905-9

Pmid:20538959

16. Barnato AE, Arnold RM, The effect of emotion and physician communication behaviors on surrogates' life-sustaining treatment decisions: a randomized simulation experiment., Crit Care Med. 2013 Jul;41(7):1686-91

Pmid:23660727

17. White DB, Ernecoff N, Buddadhumaruk P, Hong S, Weissfeld L, Curtis JR, Luce JM, Lo B, Prevalence of and Factors Related to Discordance About Prognosis Between Physicians and Surrogate Decision Makers of Critically Ill Patients., JAMA. 2016 May 17;315(19):2086-94

Pmid:27187301

18. Fried TR, Bradley EH, O'Leary J, Prognosis communication in serious illness: perceptions of older patients, caregivers, and clinicians., J Am Geriatr Soc. 2003 Oct;51(10):1398-403

Pmid:14511159

19. Chiarchiaro J, Buddadhumaruk P, Arnold RM, White DB, Quality of communication in the ICU and surrogate's understanding of prognosis., Crit Care Med. 2015 Mar;43(3):542-8

Pmid:25687030

20. Boyd EA, Lo B, Evans LR, Malvar G, Apatira L, Luce JM, White DB, "It's not just what the doctor tells me:" factors that influence surrogate decision-makers' perceptions of prognosis., Crit Care Med. 2010 May;38(5):1270-5

    Pmid:20228686

21. Verceles AC, Corwin DS, Afshar M, Friedman EB, McCurdy MT, Shanholtz C, Oakjones K, Zubrow MT, Titus J, Netzer G, Half of the family members of critically ill patients experience excessive daytime sleepiness., Intensive Care Med. 2014 Aug;40(8):1124-31

    Pmid:24898893

22. Glick DR, Motta M, Wiegand DL, Range P, Reed RM, Verceles AC, Shah NG, Netzer G, Anticipatory grief and impaired problem solving among surrogate decision makers of critically ill patients: A cross-sectional study., Intensive Crit Care Nurs. 2018 Dec;49:1-5

    Pmid:30057337

23. Pochard F, Azoulay E, Chevret S, Lemaire F, Hubert P, Canoui P, Grassin M, Zittoun R, le Gall JR, Dhainaut JF, Schlemmer B, French FAMIREA Group., Symptoms of anxiety and depression in family members of intensive care unit patients: ethical hypothesis regarding decision-making capacity., Crit Care Med. 2001 Oct;29(10):1893-7

    Pmid:11588447

24. Azoulay E, Pochard F, Kentish-Barnes N, Chevret S, Aboab J, Adrie C, Annane D, Bleichner G, Bollaert PE, Darmon M, Fassier T, Galliot R, Garrouste-Orgeas M, Goulenok C, Goldgran-Toledano D, Hayon J, Jourdain M, Kaidomar M, Laplace C, Larché J, Liotier J, Papazian L, Poisson C, Reignier J, Saidi F, Schlemmer B, FAMIREA Study Group., Risk of post-traumatic stress symptoms in family members of intensive care unit patients., Am J Respir Crit Care Med. 2005 May 1;171(9):987-94

    Pmid:15665319

25. Paulus MP, Yu AJ, Emotion and decision-making: affect-driven belief systems in anxiety and depression., Trends Cogn Sci. 2012 Sep;16(9):476-83

    Pmid:22898207

26. Turnbull AE, Hashem MD, Rabiee A, To A, Chessare CM, Needham DM, Evaluation of a strategy for enrolling the families of critically ill patients in research using limited human resources., PLoS One. 2017;12(5):e0177741

Pmid:28542632

27. Schwarzkopf D, Behrend S, Skupin H, Westermann I, Riedemann NC, Pfeifer R, Günther A, Witte OW, Reinhart K, Hartog CS, Family satisfaction in the intensive care unit: a quantitative and qualitative analysis., Intensive Care Med. 2013 Jun;39(6):1071-9

Pmid:23417207

28. Bein T, Hackner K, Zou T, Schultes S, Bösch T, Schlitt HJ, Graf BM, Olden M, Leitzmann M, Socioeconomic status, severity of disease and level of family members' care in adult surgical intensive care patients: the prospective ECSSTASI study., Intensive Care Med. 2012 Apr;38(4):612-9

Pmid:22273749

29. Bahadori K, FitzGerald JM, Risk factors of hospitalization and readmission of patients with COPD exacerbation--systematic review., Int J Chron Obstruct Pulmon Dis. 2007;2(3):241-51

Pmid:18229562

30. Quintana JM, Esteban C, Unzurrunzaga A, Garcia-Gutierrez S, Gonzalez N, Lafuente I, Bare M, de Larrea NF, Vidal S, IRYSS-COPD Group., Prognostic severity scores for patients with COPD exacerbations attending emergency departments., Int J Tuberc Lung Dis. 2014 Dec;18(12):1415-20

Pmid:25517805

31. Turnbull AE, Krall JR, Ruhl AP, Curtis JR, Halpern SD, Lau BM, Needham DM, A scenario-based, randomized trial of patient values and functional prognosis on intensivist intent to discuss withdrawing life support., Crit Care Med. 2014 Jun;42(6):1455-62

Pmid:24584065

32. Turnbull AE, Hayes MM, Brower RG, Colantuoni E, Basyal PS, White DB, Curtis JR, Needham DM, Effect of Documenting Prognosis on the Information Provided to ICU Proxies: A Randomized Trial., Crit Care Med. 2019 Jun;47(6):757-764

Pmid:30882479

33. Vasher ST, Zaeh SE, Eakin MN, Turnbull AE, Responses to a Daughter's Question about Prognosis When the Patient Is Expected to Die: A Qualitative Analysis., Ann Am Thorac Soc. 2019 Dec;16(12):1595-1598

Pmid:31419913
