# Supplementary material for: Effect of Intensivist Communication in a Simulated Setting on Interpretation of Prognosis Among Family Members of Patients at High Risk of Intensive Care Unit Admission: A Randomized Trial
Source: JAMA Netw Open. 2020 Apr 1;3(4):e201945. doi: 10.1001/jamanetworkopen.2020.1945 (PMC7113731; doi:10.1001/jamanetworkopen.2020.1945)
Supplement: Supplement 2. — eFigure 1. Definition of Difference in Belief eFigure 2. Study Flow Diagram eTable 1. Unadjusted Models of the Effect of Intensivist’s Response on Family Member Understanding of the Intensivist, Belief About Prognosis, and Difference in Belief eTable 2. Effect of Intensivist’s Response on Family Member Confidence in Their Own Prognostic Estimate and Their Perceived Prognostic Estimate of the Intensivist eTable 3. Sensitivity Analysis of Effect of Intensivist’s Response on Family Member Confidence in Their Own Prognostic Estimate and Their Perceived Prognostic Estimate of the Intensivist eReference. [file jamanetwopen-3-e201945-s002.pdf]

Supplementary Online Content

Oppenheim IM, Lee EM, Vasher ST, Zaeh SE, Hart JL, Turnbull AE. Effect of intensivist communication in a simulated setting on interpretation of prognosis among family members of patients at high risk of intensive care unit admission: a randomized trial. *JAMA Netw Open*. 2020;3(4):e201945. doi:10.1001/jamanetworkopen.2020.1945

eFigure 1. Definition of Difference in Belief

eFigure 2. Study Flow Diagram

eTable 1. Unadjusted Models of the Effect of Intensivist's Response on Family Member Understanding of the Intensivist, Belief About Prognosis, and Difference in Belief

eTable 2. Effect of Intensivist's Response on Family Member Confidence in Their Own Prognostic Estimate and Their Perceived Prognostic Estimate of the Intensivist

eTable 3. Sensitivity Analysis of Effect of Intensivist's Response on Family Member Confidence in Their Own Prognostic Estimate and Their Perceived Prognostic Estimate of the Intensivist

eReference.

This supplementary material has been provided by the authors to give readers additional information about their work.

© 2020 Oppenheim IM et al. *JAMA Network Open.*

eFigure 1. Definition of Difference in Belief

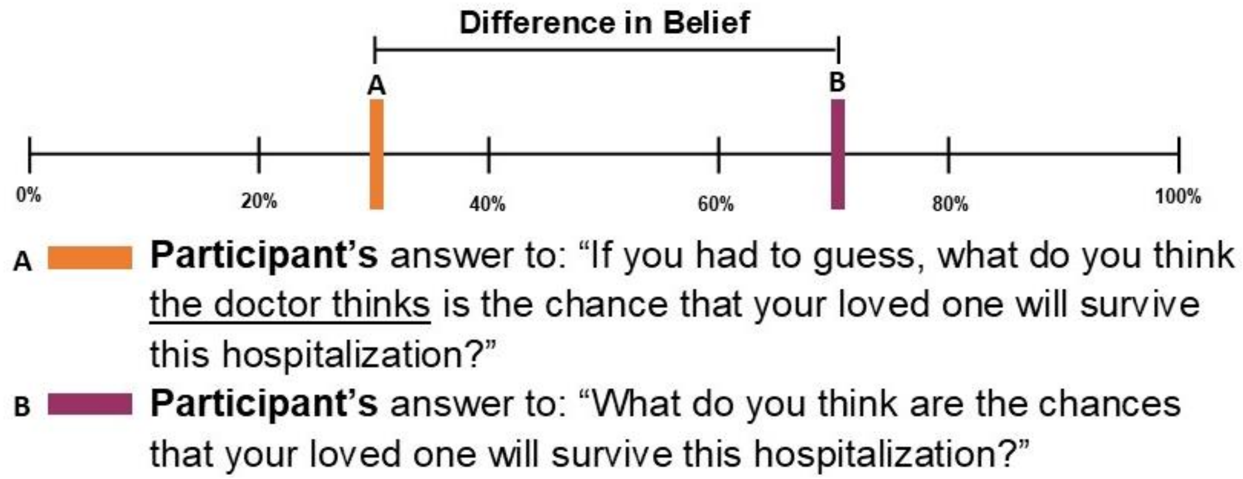

eFigure 1: Difference in Belief is defined as the participant's prognostic estimate (**B** in eFigure 1) minus their perception of the intensivist's prognostic estimate (**A** in eFigure 1).[1]

© 2020 Oppenheim IM et al. *JAMA Network Open.*

eFigure 2. Study Flow Diagram

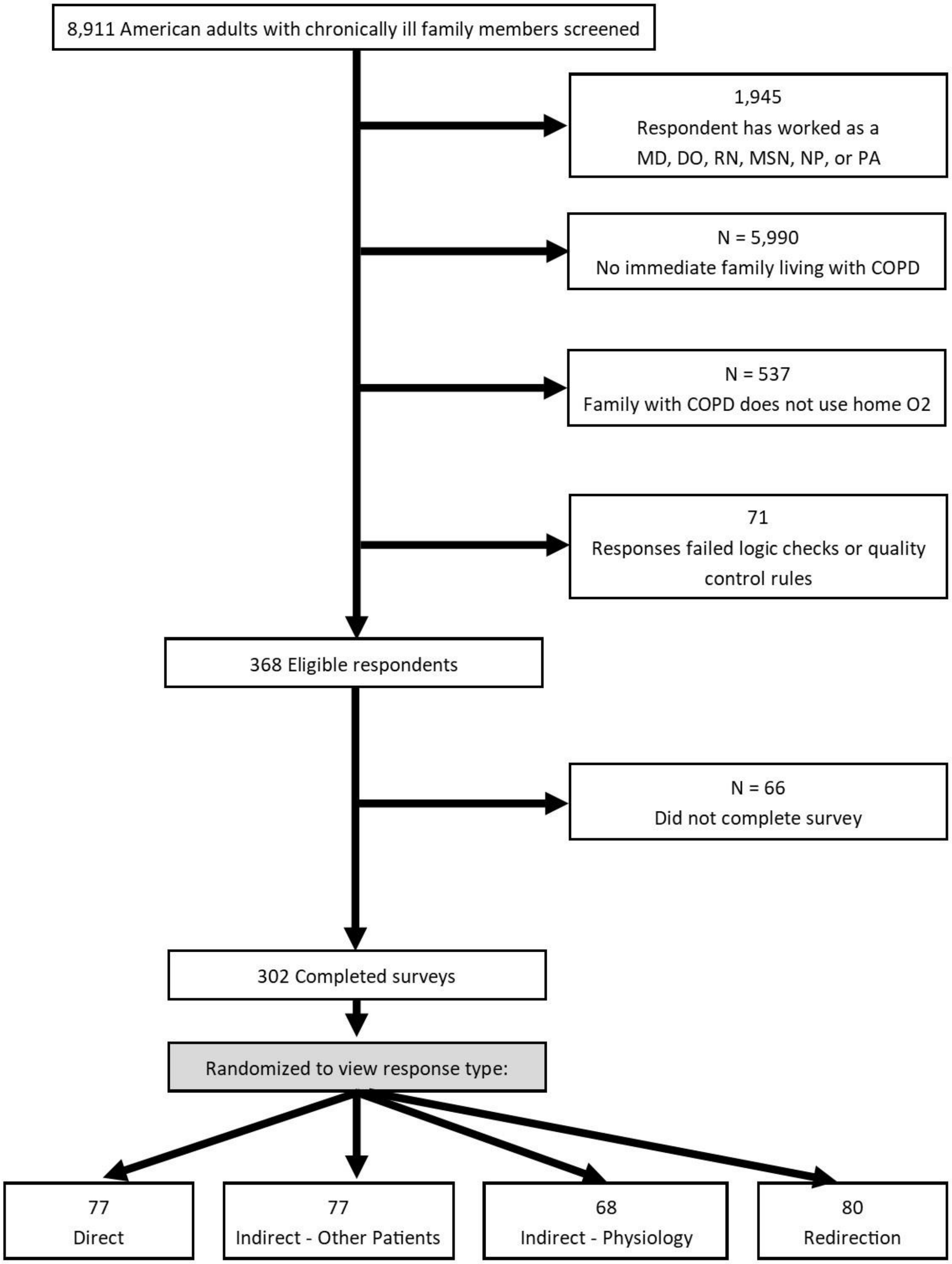

© 2020 Oppenheim IM et al. *JAMA Network Open.*

eTable 1. Unadjusted Models of the Effect of Intensivist's Response on Family Member Understanding of the Intensivist, Belief About Prognosis, and Difference in Belief

Model	β (95% CI)	P value
**Question 1:** *"What do you think* <u>*the doctor thinks*</u> *is the chance that your loved one will survive this hospitalization?"*[a]		
**Model 1**: unadjusted		
Direct	**Ref**	-
Indirect – Other Patients	10 (1, 19)	.03
Indirect – Physiology	11 (2, 21)	.02
Redirection	22 (13, 31)	<.0001
**Question 2:** *"What do* <u>*you*</u> *think are the chances that your loved one will survive this hospitalization?"*[a]		
**Model 2:** unadjusted		
Direct	**Ref**	-
Indirect – Other Patients	8 (-1, 17)	.10
Indirect – Physiology	7 (-2, 16)	.14
Redirection	17 (8, 26)	.0002
**Difference in Belief** = (response to Question 2) – (response to Question 1)		
**Model 3:** unadjusted		
Direct	**Ref**	-
Indirect – Other Patients	-2 (-9, 5)	0.54
Indirect – Physiology	-4 (-12, 3)	0.23
Redirection	-4 (-11, 3)	0.22

a. Measured on a 0 – 100 scale, with higher scores indicating a greater likelihood of survival.
Abbreviations: CI, confidence interval; Ref, reference

© 2020 Oppenheim IM et al. *JAMA Network Open.*

eTable 2. Effect of Intensivist's Response on Family Member Confidence in Their Own Prognostic Estimate and Their Perceived Prognostic Estimate of the Intensivist

	Intensivist's Response					
	**Direct (n=77)**	**Indirect – Other patients (n=77)**	**Indirect – Physiology (n=68)**	**Redirection (n=80)**	**Difference in proportions[c] (95% CI)**	**P value**
Confident they know what the doctor thinks[a] n (%)	52 (68%)	48 (62%)	–	–	.05 (-.11, .21)	0.61
		–	43 (63%)	–	.04 (-.13, .21)	0.71
		–	–	51 (64%)	.04 (-.12, .20)	0.74
Confident in their own understanding[b] n (%)	59 (77%)	50 (65%)	–	–	.12 (-.04, .27)	0.16
		–	53 (78%)	–	-.01 (-.16, .14)	1.00
		–	–	58 (73%)	.04 (-.11, .19)	0.68

a. Response to the question *"How confident are you that you know what the doctor thinks your loved one's chances for surviving the hospitalization are?"* using on a 5-point Likert scale, with higher scores indicating greater confidence. Responses scored a 4 or 5 were analyzed as "Confident."
b. Response to the question *"How confident are you that you understand your loved one's chances for surviving the hospitalization?"* using on a 5-point Likert scale, with higher scores indicating greater confidence. Responses scored a 4 or 5 were analyzed as "Confident."
c. The difference in the proportion of respondents who are confident compared to those randomized to view a direct response to the question.
Abbreviations: CI, confidence interval; Ref, reference

© 2020 Oppenheim IM et al. *JAMA Network Open.*

eTable 3. Sensitivity Analysis of Effect of Intensivist's Response on Family Member Confidence in Their Own Prognostic Estimate and Their Perceived Prognostic Estimate of the Intensivist

	Intensivist's Response					
	**Direct (n=77)**	**Indirect – Other patients (n=77)**	**Indirect – Physiology (n=68)**	**Redirection (n=80)**	**Difference in proportions[c] (95% CI)**	**P value**
Confident they know what the doctor thinks[a] n (%)	65 (84%)	61 (79%)	–	–	.05 (-.08, .19)	0.53
		–	56 (73%)	–	.02 (-.11, .16)	0.91
		–	–	64 (80%)	.04 (-.02, .18)	0.61
Confident in their own understanding[b] n (%)	68 (88%)	62 (81%)	–	–	.08 (-.05, .20)	0.27
		–	59 (87%)	–	-.02 (-.11, .14)	0.98
		–	–	70 (88%)	.01 (-.10, .12)	1.00

a. Response to the question *"How confident are you that you know* *what the doctor thinks* *your loved one's chances for surviving the hospitalization are?"* using on a 5-point Likert scale, with higher scores indicating greater confidence. Responses scored 3, 4, or 5 were analyzed as "Confident."
b. Response to the question *"How confident are you that you understand your loved one's chances for surviving the hospitalization?"* using on a 5-point Likert scale, with higher scores indicating greater confidence. Responses scored 3, 4, or 5 were analyzed as "Confident."
c. The difference in the proportion of respondents who are confident compared to those randomized to view a direct response to the question.
Abbreviations: CI, confidence interval

© 2020 Oppenheim IM et al. *JAMA Network Open.*

eReference.

1. White DB, Ernecoff N, Buddadhumaruk P, et al. Prevalence of and Factors Related to Discordance About Prognosis Between Physicians and Surrogate Decision Makers of Critically Ill Patients. JAMA. 2016;315(19):2086. doi:10.1001/jama.2016.5351

© 2020 Oppenheim IM et al. *JAMA Network Open.*
