# Supplementary material for: Effect of Intensivist Communication in a Simulated Setting on Interpretation of Prognosis Among Family Members of Patients at High Risk of Intensive Care Unit Admission: A Randomized Trial
Source: JAMA Netw Open. 2020 Apr 1;3(4):e201945. doi: 10.1001/jamanetworkopen.2020.1945 (PMC7113731; doi:10.1001/jamanetworkopen.2020.1945)
Supplement: Supplement 3. — Data Sharing Statement [file jamanetwopen-3-e201945-s003.pdf]

Data Sharing Statement

Oppenheim. Effect of Intensivist Communication in a Simulated Setting on Interpretation of Prognosis Among Family Members of Patients at High Risk of Intensive Care Unit Admission. JAMA Netw Open. Published April 01, 2020. 10.1001/jamanetworkopen.2020.1945

Data
Data available: Yes
Data types: Deidentified participant data
How to access data: https://doi.org/10.7281/T1/VWQ4J3
When available: beginning date: 04-01-2020

Supporting Documents
Document types: Codebook, README, R project and markdown files containing code for all analyses and figures. Documents available as .csv, .txt, .Rproj, and .Rmd files.

Additional Information
Who can access the data: Anyone
Types of analyses: Any
Mechanisms of data availability: Freely available for download at:

Oppenheim, IM; Lee, EM; Vasher, ST; Zaeh, SE; Hart, JL; Turnbull, AE, 2020, "Data associated with the 2020 JAMA Network Open publication entitled: ‘Effect of Intensivist Communication in a Simulated Setting on Interpretation of Prognosis Among Family Members of Patients at High Risk for Intensive Care Unit Admission: A Randomized Trial'", https://doi.org/10.7281/T1/VWQ4J3, Johns Hopkins University Data Archive.
